# HIV reservoirs are dominated by genetically younger and clonally enriched proviruses

**DOI:** 10.1128/mbio.02417-23

**Published:** 2023-11-16

**Authors:** Natalie N. Kinloch, Aniqa Shahid, Winnie Dong, Don Kirkby, Bradley R. Jones, Charlotte J. Beelen, Daniel MacMillan, Guinevere Q. Lee, Talia M. Mota, Hanwei Sudderuddin, Evan Barad, Marianne Harris, Chanson J. Brumme, R. Brad Jones, Mark A. Brockman, Jeffrey B. Joy, Zabrina L. Brumme

**Affiliations:** 1 Faculty of Health Sciences, Simon Fraser University, Burnaby, British Columbia, Canada; 2 British Columbia Centre for Excellence in HIV/AIDS, Vancouver, British Columbia, Canada; 3 Bioinformatics Program, University of British Columbia, Vancouver, British Columbia, Canada; 4 Infectious Diseases Division, Department of Medicine, Weill Cornell Medical College, New York, New York, USA; 5 Experimental Medicine Program, University of British Columbia, Vancouver, British Columbia, Canada; 6 Department of Family Practice, Faculty of Medicine, University of British Columbia, Vancouver, British Columbia, Canada; 7 Department of Medicine, University of British Columbia, Vancouver, British Columbia, Canada; 8 Department of Molecular Biology and Biochemistry, Faculty of Science, Simon Fraser University, Burnaby, British Columbia, Canada; Medical School, National and Kapodistrian University of Athens, Athens, Greece

**Keywords:** HIV reservoir, persistence, genomic integrity, molecular dating, proviral landscape, phylogenetics, low-level viremia, rebound

## Abstract

In order to cure human immunodeficiency virus (HIV), we need to better understand the within-host evolutionary origins of the small reservoir of genome-intact proviruses that persist within infected cells during antiretroviral therapy (ART). Most prior studies on reservoir evolutionary dynamics, however, did not discriminate genome-intact proviruses from the vast background of defective ones. We reconstructed within-host pre-ART HIV evolutionary histories in six individuals and leveraged this information to infer the ages of intact and defective proviruses sampled after an average of >9 years on ART, along with the ages of rebound and low-level/isolated viremia occurring during this time. We observed that the longest-lived proviruses persisting on ART were exclusively defective, usually due to large deletions. In contrast, intact proviruses and rebound HIV exclusively dated to the years immediately preceding ART. These observations are consistent with genome-intact proviruses having shorter lifespans, likely due to the cumulative risk of elimination following viral reactivation and protein production. Consistent with this, intact proviruses (and those with packaging signal defects) were three times more likely to be genetically identical compared to other proviral types, highlighting clonal expansion as particularly important in ensuring their survival. By contrast, low-level/isolated viremia sequences were heterogeneous in terms of age, with some potentially originating from defective proviruses. Results reveal that the HIV reservoir is dominated by clonally enriched and genetically younger sequences that date to the period of untreated infection when viral populations had been under within-host selection pressures for the longest duration. Knowledge of these qualities may help focus strategies for reservoir elimination.

**IMPORTANCE:**

Characterizing the human immunodeficiency virus (HIV) reservoir that endures despite antiretroviral therapy (ART) is critical to cure efforts. We observed that the oldest proviruses persisting during ART were exclusively defective, while intact proviruses (and rebound HIV) dated to nearer ART initiation. This helps explain why studies that sampled sub-genomic proviruses on-ART (which are largely defective) routinely found sequences dating to early infection, whereas those that sampled replication-competent HIV found almost none. Together with our findings that intact proviruses were more likely to be clonal, and that on-ART low-level/isolated viremia originated from proviruses of varying ages (including possibly defective ones), our observations indicate that (i) on-ART and rebound viremia can have distinct within-host origins, (ii) intact proviruses have shorter lifespans than grossly defective ones and thus depend more heavily on clonal expansion for persistence, and (iii) an HIV reservoir predominantly “dating” to near ART initiation will be substantially adapted to within-host pressures, complicating immune-based cure strategies.

## INTRODUCTION

Following infection, human immunodeficiency virus 1 (HIV-1) integrates its genome into that of the host cell, usually a CD4^+^ T-lymphocyte (1, 2). Most infected cells die or are eliminated by the immune system (3), usually within 2 days of infection (4), but a minority persist even during long-term antiretroviral therapy (ART) and can fuel viral rebound if ART is interrupted (5–8). If we are to cure HIV, it is critical to understand the within-host evolutionary origins and dynamics of the genome-intact and replication-competent proviruses that comprise this persistent HIV reservoir.

Seeding of HIV sequences into the reservoir begins immediately following infection (9–12) and continues until ART initiation (13–16). During untreated infection, reservoir turnover is relatively rapid (14, 16–18): recent half-life estimates are on the order of half a year, compared to nearly 4 years for intact proviruses during the initial years of ART (19–27). As such, if ART is not initiated until chronic infection, many early within-host HIV lineages will have already been eliminated by this time. This is supported by the frequent observation that most proviruses that persist during ART originally integrated in the year or two prior to ART initiation (13–16, 28, 29). Nevertheless, proviruses older than this, some dating as far back as transmission, are also routinely recovered during ART (13–16). While it is now clear that host cell features, such as genomic integration site (30–37) and clonal expansion (21, 31, 33, 38–47), influence how long an HIV provirus will persist within-host, the contribution of viral genetic features to proviral persistence remains incompletely understood.

Strong evidence nevertheless supports such a relationship. Most notably, genetically intact proviruses decay more rapidly during ART than defective ones, which harbor large deletions, hypermutation, packaging signal region defects, point mutations, and/or other defects (21, 22, 25–27, 38, 48, 49). This more rapid decay explains why intact proviruses constitute only ~5% of the proviral pool during long-term therapy (48–50). Assuming that intact proviruses also decay more rapidly during untreated infection, one would predict that, in individuals who did not initiate ART until chronic infection, the oldest persisting proviruses (i.e., those dating to early infection) would primarily be defective, whereas intact proviruses would generally be “younger” (i.e., date to the year or two before ART initiation). No studies to our knowledge have leveraged information from pre-ART within-host evolutionary histories to elucidate the integration dates of both intact and defective proviruses sampled during ART (13–16, 28, 29). All but one prior study collected sub-genomic sequences, which meant that intact proviruses could not be distinguished from defective ones (48, 49), while the remaining study exclusively sampled replication-competent HIV following *ex vivo* stimulation and, therefore, could not investigate age differences between intact and defective sequences (29).

Our knowledge of the within-host evolutionary origins of HIV sequences reactivated from the reservoir *in vivo*—namely, HIV RNA sequences rebounding in plasma following ART interruption, as well as low-level or isolated viremia occurring during otherwise suppressive therapy—also remains limited. While rebound HIV presumably originates from genome-intact proviruses, on-ART viremia can originate from both intact (51) and defective proviruses (52). Indeed, as we now appreciate that some defective proviruses can produce HIV transcripts, proteins (50, 53), and even virions (52), and that cells harboring them can, in some cases, be recognized by the immune system (53, 54), it is important to better understand the longevity of defective proviruses as well. To address these knowledge gaps, we used phylogenetic methods (13) to infer the age distributions of intact proviruses, different types of defective proviruses, and reactivated HIV sequences (both *in vivo* and *ex vivo*) from six individuals who had been receiving ART for a median of 9 years.

## RESULTS

### Participant characteristics and reservoir sampling

**Fig 1:**
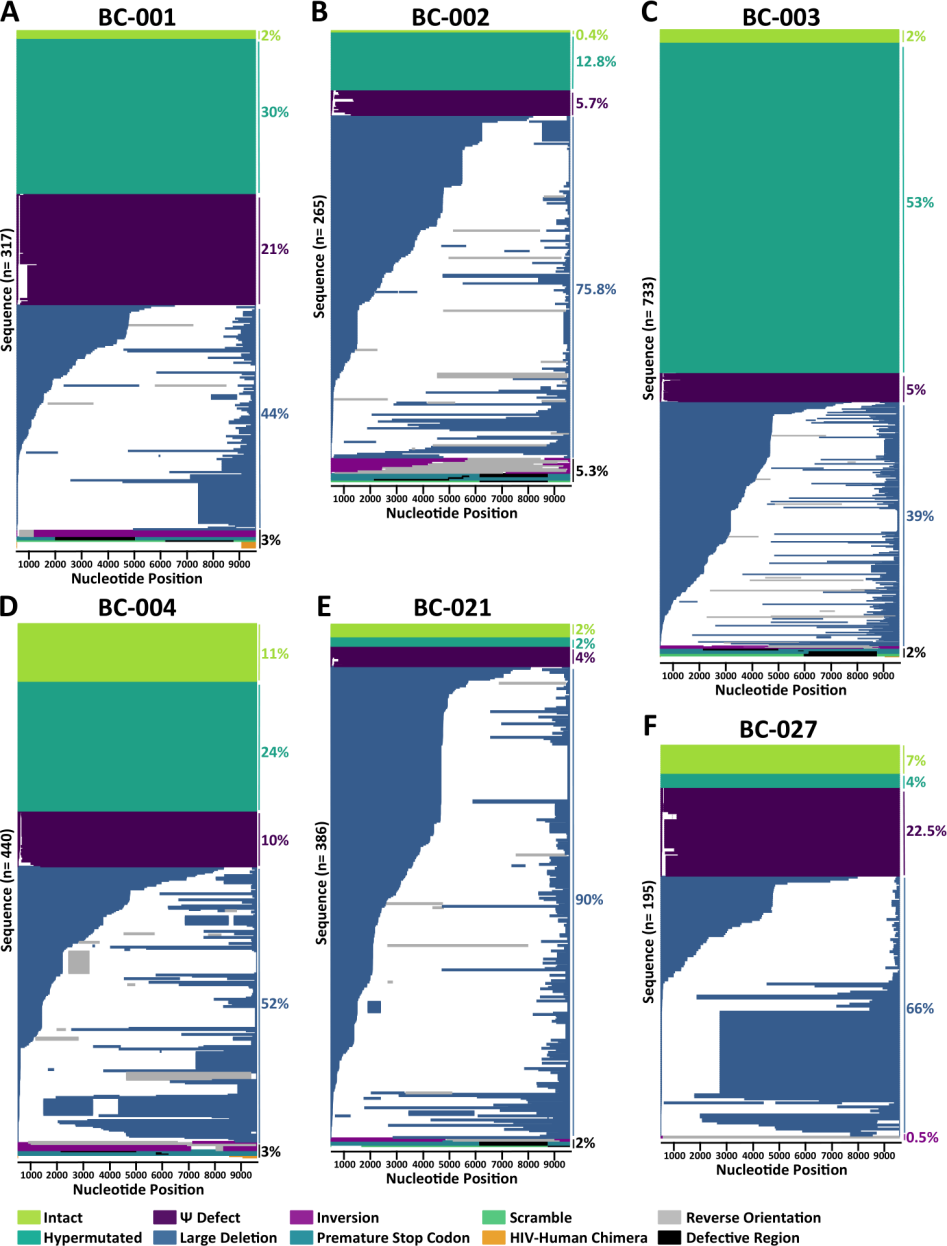
Participant proviral landscapes. The individual panels depict the near-full-length proviral genomes isolated from each of the six participants, where proviruses are colored by genomic integrity as described in the pictorial legend (note: white denotes deletions; gray denotes reverse orientation; and black denotes defective genes). The total number of proviral sequences is shown at the left of each plot, with the frequencies of each sequence type shown at the right.

We isolated 2,336 near-full-length proviruses (median 352; range 195–733 per participant) at a single time point from six participants living with HIV who had been receiving ART for a median of 8.9 (range 7.2–12.2) years (Table 1; Fig. 1). For four of these participants (BC-001 through BC-004), sub-genomic proviral sequencing had previously been performed (13, 28), but these data were not incorporated into the present study because genome integrity could not be established (13, 28). Here, we used a strict definition of genome-intact that required all HIV proteins (including accessory proteins) to be intact, an approach that differs from some previous studies (21, 23, 40). Moreover, as we retained only amplicons that were sequenced end-to-end (see Materials and Methods), we could definitively classify each provirus as intact or defective (i.e., there was no “inferred intact” category). For four participants, we performed additional on-ART sampling as follows: from BC-003 and BC-004, we isolated two and seven full-genome HIV RNA sequences, respectively, from limiting-dilution quantitative viral outgrowth assays (QVOA) performed on the same sample as the proviral sequencing. From BC-001, we isolated nine subgenomic (*nef*) HIV RNA sequences from plasma after ART interruption. From BC-001, BC-002, and BC-004, we isolated 104 HIV RNA sequences from low-level or isolated viremia events during otherwise suppressive ART. We defined these as isolated or intermittent viremia generally below 1,000 HIV RNA copies/mL according to WHO guidelines (55), though participants 2 and 4 had isolated measurements exceeding this, but with no record of ART interruption nor the appearance of new antiretroviral resistance mutations.

**TABLE 1:** Participant characteristics and HIV sequences collected

Participant	Years from estimated infection^*a*^ to first ART^*b*^	Years of pre-ART sampling (*n* time points)	Number of pre-ART HIV RNA sequences collected (% unique)	Subsequent years of ART	Number of proviruses collected on ART (% unique)	Number of intact proviruses collected (% unique)	Number of QVOA sequences collected on ART (% unique)	Number of rebound HIV RNA sequences collected (% unique)	Number of low-level viremia sequences collected (% unique)
BC-001	11.9	10 (15)	102 (91)	8	317 (65)	5 (100)	–	9 (56)	1 (100)
BC-002	14.6	9.5 (17)	160 (82)	9.6	265 (75)	1 (100)	–	–	13 (77)
BC-003	5.9	4.75 (8)	122 (93)	8.7	733 (88)	15 (87)	2 (100)	–	–
BC-004	2.2	0.75 (4)	65 (91)	9.1	440 (69)	47 (53)	7 (100)	–	90 (9)
BC-021	5	2.5 (8)	221 (79)	12.2	386 (72)	9 (44)	–	–	–
BC-027	26.5	14.25 (7)	215 (82)	7.2	195 (54)	14 (43)	–	–	–

^*a*^ Estimated using clinical records (where available), participant-reported date, or the mean root date of within-host phylogenies, whichever was earliest.

^*b*^ First treatment with at least triple ART.

We also isolated 885 HIV RNA *nef* sequences (median 141; range 65–221 per participant) from a median of 8 (range 4–17) longitudinal archived plasma samples that spanned a median of 7.1 (range 0.75–14.25) years prior to ART (Table 1). These sequences were used to reconstruct participants’ pre-ART HIV evolutionary histories, which were in turn used to infer the ages of HIV sequences persisting on ART. We used *nef* because it evolves rapidly within-host but is nevertheless representative of within-host HIV diversity elsewhere in the genome (13). Using *nef* also allowed us to maximize the number of sequences whose integration dates could be inferred, as *nef* is the most likely region to be intact in proviruses persisting on ART (48). All participants had HIV subtype B, and within-host sequences were monophyletic with no evidence of superinfection (Fig. S1 and S2).

### Proviral landscape during long-term ART

Of the 2,336 near-full-length proviruses collected, only 4% were genome-intact: a median of 2% of sequences (range 0.4%–11%; 1–47 sequences) per participant (Fig. 1 and Table 1; Table S1). BC-004 had the highest proportion of intact sequences, at 11% (*n* = 47; 25 unique), while BC-002 had the lowest, at 0.4% (*n* = 1) (Fig. 1B and D, Table 1; Table S1). For four participants (BC-001, BC-002, BC-003, and BC-021), the recovered proportion of intact proviruses was lower than that predicted by the Intact Proviral DNA Assay (IPDA) (Table S1), which is not surprising given the inefficiency of long-range PCR (56) and the ability of sequencing to capture defects outside the IPDA target regions (48). Proviruses with large deletions dominated in all participants except BC-003, and made up a median of 59% (range of 39%–90%) of proviruses per participant (Fig. 1A through F; Table S1). These varied greatly in length—the shortest, recovered from BC-004, was just 167 base pairs—and many had additional defects such as gene inversions, scrambles, and hypermutation. We also observed evidence of template switching between repeated genomic elements during reverse transcription as a reproducible mechanism for large deletion formation, both within and between individuals. Thirteen distinct sequences from BC-003, for example, had a deletion spanning HIV genomic nucleotides 4,781–9,064 (numbering according to the HXB2 reference strain), where the sequence flanking the deletion was “TTTTAAAAGAAAAGGGGGGA.” These exact breakpoints, which have been described by others (38, 49), were also observed in one of BC-001’s proviruses, one of BC-004’s, and 11 distinct proviruses from BC-021.

By contrast, hypermutation dominated BC-003’s proviral landscape, at 53%. Prior CD4^+^ T-cell phenotyping of this participant had revealed a 47% frequency of naive CD4^+^ T-cells (28), which have been shown to be enriched in hypermutated proviruses (57). On average, however, hypermutated proviruses comprised a median of 19% (range 2%–53%) of participants’ proviral pools, whereas those with packaging signal defects comprised a median of 8% (range 4%–23%). Proviruses with inversions, gene scrambles, premature stop codons, and HIV-human chimeras were uncommon, making up a median of 2% (range 0.5%–5%) of proviruses per participant.

As expected (48), *nef* was the most commonly intact region in four participants (BC-001, BC-002, BC-004, and BC-027) and the second most commonly intact region in BC-003 and BC-021 (for whom *gag* was the most commonly intact). Other frequently intact regions varied markedly by participant but included *vpr* (BC-001), *vif* (BC-004), and *vpu* (BC-027). The fraction of proviruses with an intact *nef* region ranged from 65% (127 sequences) for BC-027 to only 16% (115 sequences) for BC-003, due to the high proportion of hypermutated proviruses in the latter participant. The subset of *nef*-intact proviruses was those whose integration dates could be phylogenetically inferred, as described below.

### Proviral clonality during long-term ART

**Fig 2:**
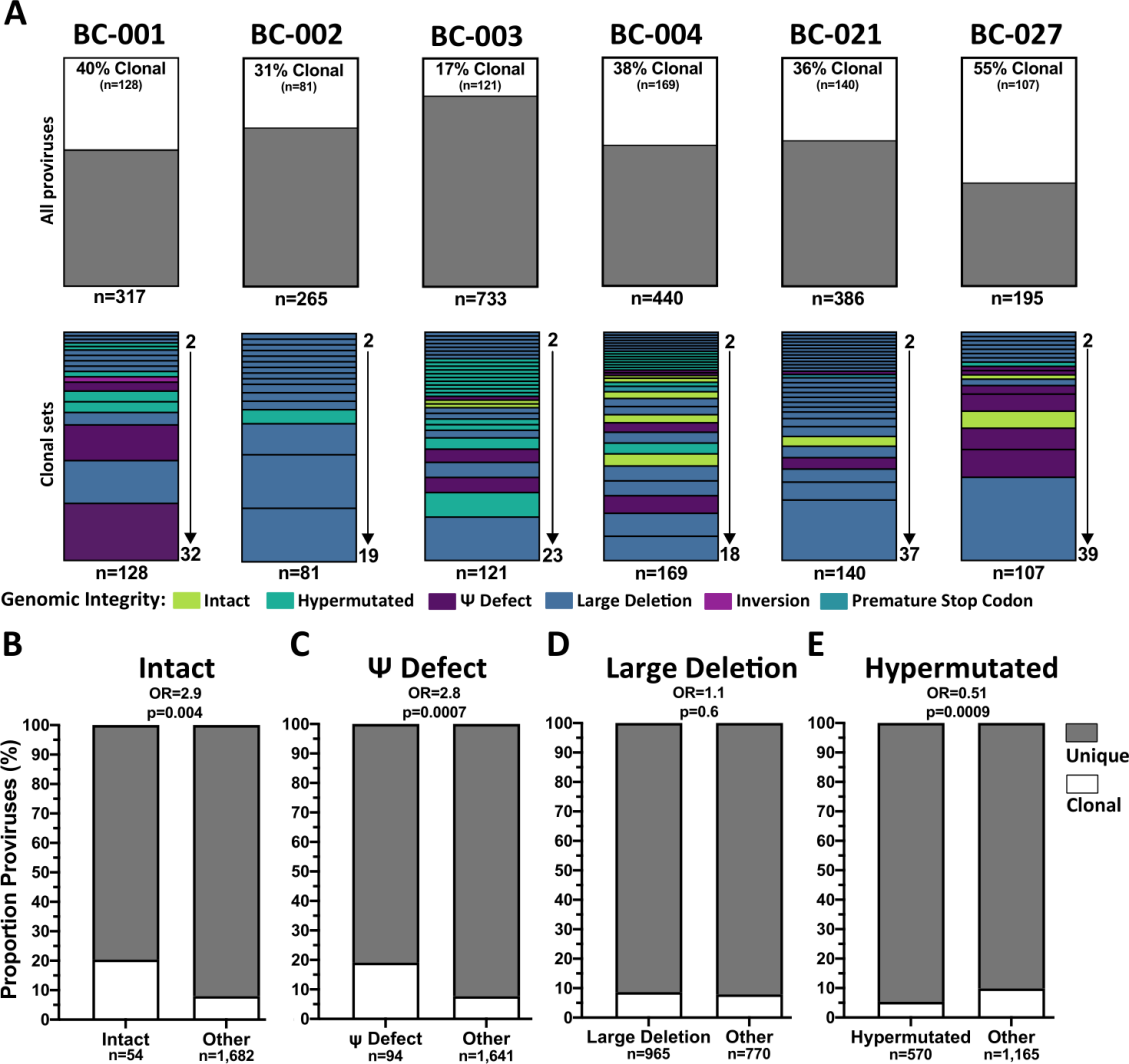
Proviral sequence clonality and relationship with genomic integrity. (A) (Top) Proportion of clonal sequences (those observed more than once; white) versus those observed only once (gray), in each participant. (Bottom) Breakdown of clonal sequences by clone size (bar height) and genomic integrity (color). (B–E) Proportion of clonal sequences within each group of interest, compared to all other proviruses. The proportion of clonal sequences is in white, with unique sequences in gray. *P*-values are computed using Fisher’s exact test and are not corrected for multiple comparisons.

Consistent with the major role of clonal expansion in sustaining the HIV reservoir (21, 31–33, 38–42, 44, 58), a median of 39% (range 17%–55%) of proviruses were identical (100% sequence identity) to at least one other from that participant, where an average of 24 such “clonal sets” (range 16–33) were recovered per participant (Fig. 2A, top). BC-003 had the lowest proportion of clonal sequences, at 17% (*n* = 121), while BC-027 had the highest, at 55% (*n* = 107). BC-027 also harbored the most abundant clone: isolated 39 times, it harbored a ~3,000 base deletion and made up 20% of the proviral pool (Fig. 2A, bottom). BC-001’s three most frequent clones, which were recovered between 20 and 32 times each and included two sequences with packaging signal defects, together made up 24% of the proviral pool.

Clonal frequency differed significantly by proviral genomic integrity (Fig. 2B through E). Across all participants, intact proviruses were nearly three times more likely to be part of a clonal set (19% of intact compared to 8% of other proviruses were clonal; odds ratio [OR] 2.9; *P* = 0.004, Fig. 2B), as were proviruses with packaging signal defects (OR 2.8; *P* = 0.0007; Fig. 2C). Proviruses with large deletions were not preferentially clonal (*P* = 0.6; Fig. 2D), and hypermutated proviruses were less likely to be clonal (OR 0.51; *P* = 0.0009, Fig. 2E). These observations remained consistent when we repeatedly subsampled sequences from the comparison group to the same depth as the query group (Fig. S3).

### Elucidating the ages of intact and defective proviruses, and reservoir-origin viremia

We used a phylogenetic approach (13) to estimate the ages of HIV sequences persisting on ART. To do this, we inferred within-host phylogenies relating longitudinal pre-ART plasma HIV RNA *nef* sequences with viral sequences sampled post-ART, whose integration dates we wished to estimate. The latter included near-full-length proviruses for all participants, as well as QVOA outgrowth sequences, HIV RNA from on-ART viremia episodes, and/or HIV RNA isolated after ART interruption for four participants. To mitigate the inherent uncertainty in within-host phylogenetic reconstruction, we inferred distributions of 1,500–6,000 phylogenies per participant using Bayesian approaches, and conditioned results over all trees.

We began by rooting each tree at the location that maximizes the correlation between the root-to-tip distances and sampling dates of the pre-ART plasma HIV RNA sequences (see Materials and Methods). For data sets that retain sequences from early infection (either pre-ART plasma or older proviruses), the root should represent the transmitted/founder virus or a very close descendant thereof. For data sets that do not retain any early sequences, the root will represent a more distant descendant of the founder. Root placement is key, as it is not possible to “date” sequences to earlier than this point.

We then fit a linear model relating the root-to-tip genetic distances of unique pre-ART plasma HIV RNA *nef* sequences to their sampling dates. The slope of this line, which represents the average within-host pre-ART *nef* evolutionary rate, was then used to convert the root-to-tip distance of each post-ART sequence of interest to its integration date. We conditioned these dates over all trees that met our quality control criteria (see Materials and Methods), yielding integration date point estimates and 95% highest posterior density (HPD) intervals for each sequence. As trees were inferred from *nef* sequences, only *nef*-intact proviruses could be dated: this amounted to a median of 121 (range 70–165) proviruses per participant, representing a median of 32% (range 16%–65%) of all proviruses collected.

#### Participant BC-001

**Fig 3:**
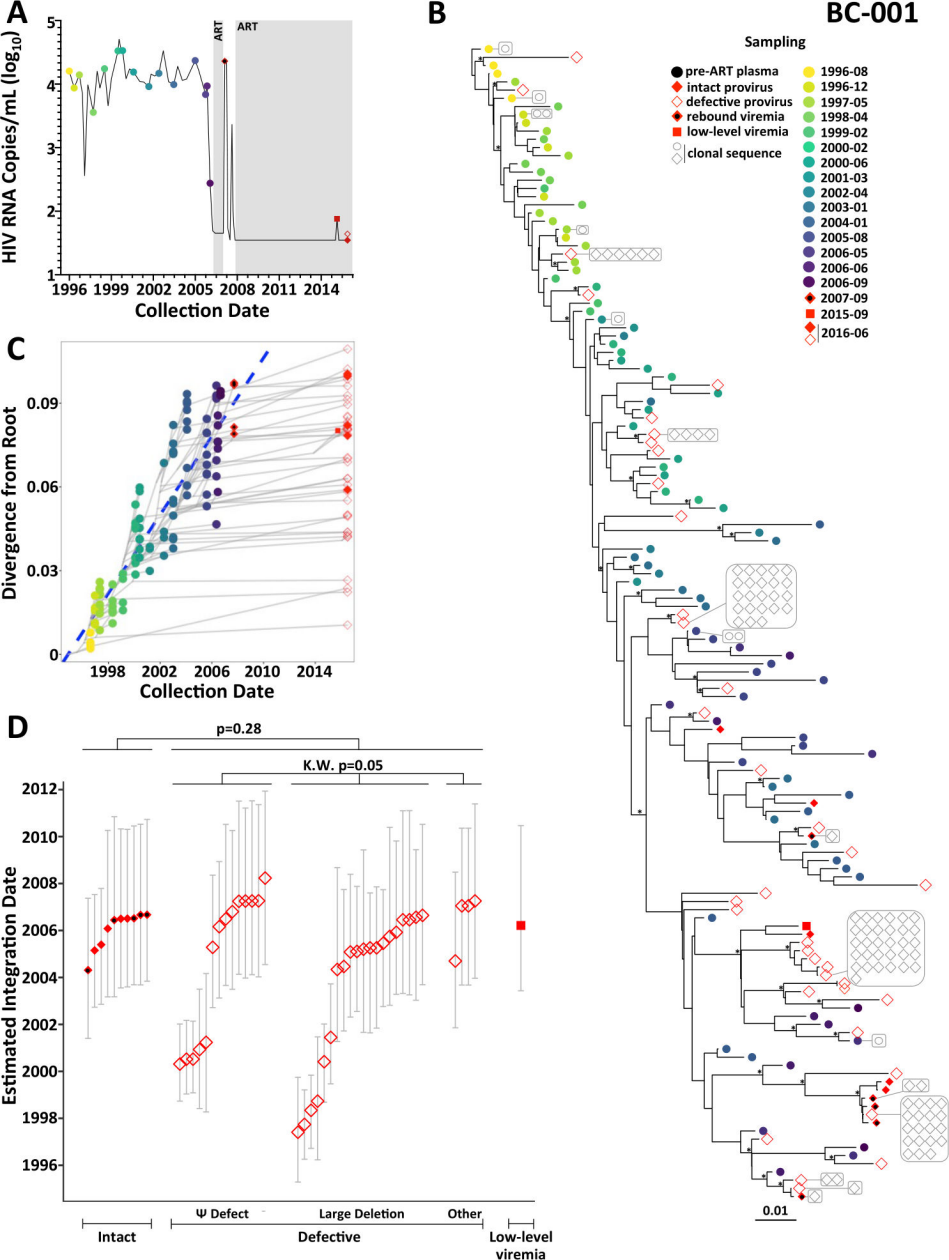
Within-host HIV evolutionary reconstruction and integration date inference of on-ART sequences for participant BC-001. (A) Participant clinical and sampling history. Plasma viral load is shown as a solid black line, with pre-ART plasma HIV RNA sampling dates shown as colored circles. Shaded periods denote ART. Red symbols denote sampling dates of on-ART sequences of interest, including intact proviruses (solid red diamonds), defective proviruses (open red diamonds), plasma rebound viremia following ART interruption (solid red diamonds with a black dot), and on-ART low-level viremia (solid red squares). (B) Example within-host phylogeny, which is the highest likelihood tree derived from Bayesian inference, rooted at the most recent common ancestor as described in Materials and Methods. Scale in estimated substitutions per nucleotide site. Asterisks identify nodes supported by posterior probabilities ≥ 70%. Open gray symbols indicate clonal sequences, which are boxed together and are connected by a gray line to their representative in the phylogeny. (C) HIV sequence divergence-versus-time plot. The blue dashed regression line represents the linear model relating the root-to-tip distances of distinct pre-ART plasma HIV RNA sequences (colored circles) to their sampling times. This regression line is used to convert the root-to-tip distances of distinct proviral sequences sampled during ART (red symbols) to their integration dates. Light gray lines denote the ancestral relationships between HIV sequences. Sequences from the final pre-ART timepoint were included in the phylogeny but excluded from the regression, as the participant had initiated ART and viral load was decreasing at this time. (D) Integration date point estimates and 95% highest posterior density intervals for distinct proviral and plasma rebound sequences recovered from this participant, stratified by sequence type. *P*-values above the plot compare the integration date point estimates between groups: the Mann-Whitney *U* test was used for between-group comparisons (e.g., all intact versus all defective), while the Kruskal-Wallis (K.W.) test was used to compare the three different types of defective proviruses.

Participant BC-001 was diagnosed with HIV in August 1996. ART was initiated in August 2006 but interrupted shortly thereafter, and durable viral suppression was not achieved until June 2008 (Fig. 3A). We collected 102 plasma HIV RNA *nef* sequences from 15 pre-ART time points spanning 10 years, along with 317 proviruses sampled in June 2016, ~8 years after ART initiation, 131 (41%) of which had an intact *nef* (Fig. 3A, Table 1). We also isolated nine (five unique) HIV RNA *nef* sequences from plasma collected in September 2007, after ART was interrupted and viremia rebounded to 23,000 copies/mL, and one HIV RNA *nef* sequence from plasma collected in September 2015 when an isolated viremia “blip” to 76 copies/mL occurred. All 1,500 rooted within-host phylogenies showed strong molecular clock signal, yielding an average estimated *nef* pre-ART evolutionary rate of 3 × 10^−5^ (95% HPD 1.7 × 10^−5^–4.2 × 10^−5^) substitutions/nucleotide site/day, and a mean root date of February 1995 (95% HPD December 1993–February 1996), approximately 18 months prior to the participant’s HIV diagnosis. Details of the phylogenetic inference, including substitution rates and root dates, are shown in Table S2; an example phylogeny and linear model are shown in Fig. 3B and C, respectively; and an amino acid highlighter plot is shown in Fig. S4A. The phylogeny exhibited the ladder-like form typical of within-host HIV evolution, which is characterized by serial genetic bottlenecks imposed by host immune pressures (59–62), a phenomenon that is apparent in the selective sweeps occurring at numerous Nef residues during untreated infection (Fig. S4A).

The unique HIV sequences sampled post-ART interspersed throughout the phylogeny, consistent with their continual archiving throughout infection. Averaging their phylogenetically-derived integration dates across all 1,500 trees revealed that the oldest of these sequences, a provirus with a large deletion, was estimated to have integrated in May 1997, 19 years prior to sampling. The youngest provirus was estimated to have integrated in March 2008, during the ART interruption, consistent with reservoir re-seeding during this rebound event. Despite this substantial spread in integration dates, ~50% of proviruses persisting during long-term ART dated to within 1.25 years of ART initiation (Fig. 3D) and, therefore, harbored accumulated mutational adaptations to within-host pressures (Fig. S4A).

Notably, all of the sequences sampled on ART that were estimated to have integrated in the first 5 years of infection (i.e., in ~2001 or prior), were defective proviruses (Fig. 3D). By contrast, all intact proviruses, as well as the HIV RNA sequences from the 2007 viremia rebound, were estimated to have integrated in the 2.75 years prior to ART. Though this is consistent with the hypothesis that intact HIV sequences persisting on ART are overall younger than the larger defective proviral pool, this comparison did not reach statistical significance (*P* = 0.28; Fig. 3D), as many defective proviruses also dated to this period. Integration dates did not significantly differ between defective provirus types (Kruskal-Wallis *P* = 0.05), though the very oldest all harbored large deletions. The single sequence recovered from the 2015 isolated viremia event dated to March 2006, close to ART initiation.

#### Participant BC-002

**Fig 4:**
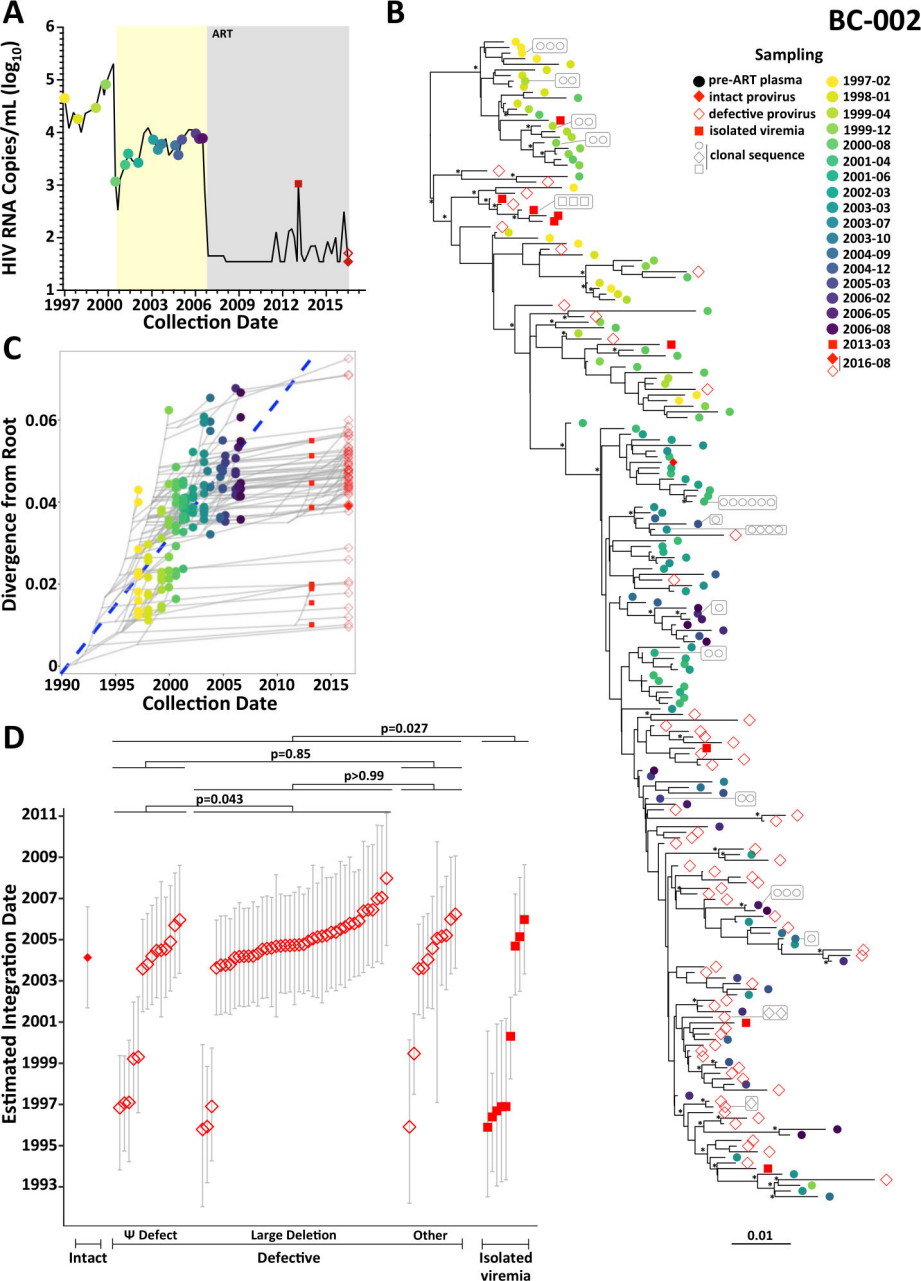
Within-host HIV evolutionary reconstruction and integration date inference of on-ART sequences for participant BC-002. The legend is the same as Fig. 3, except for the following modifications. For participant BC-002, only intact proviruses (solid red diamonds), defective proviruses (open red diamonds) and isolated on-ART plasma viremia sequences (solid red squares) were dated. In panel A, the yellow shading denotes the period of non-suppressive dual ART. In panel D, the Kruskal-Wallis test comparing the three types of defective proviruses was significant, so all statistically significant *P*-values from the pairwise post-test comparisons, corrected for multiple comparisons, are shown.

Participant BC-002 was diagnosed with HIV in April 1995 and received non-suppressive dual ART between July 2000 and December 2006, after which viral suppression was finally achieved on triple ART (Fig. 4A). Viral suppression was maintained until May 2011, after which frequent low-level viremia occurred, reaching a peak of 1,063 HIV RNA copies/mL in March 2013, despite no documented ART interruption during this time. We collected 160 plasma HIV RNA *nef* sequences from 17 time points spanning 9.5 years pre-ART (Fig. 4A, Table 1), along with 265 proviruses sampled in August 2016, 9.6 years after ART initiation, 70 (26%) of which were *nef*-intact. We also isolated 13 (10 unique) plasma HIV RNA *nef* sequences from 2013 during on-ART viremia. All 1,500 within-host phylogenies exhibited strong molecular clock signal, yielding a mean pre-ART *nef* evolutionary rate of 1.5 × 10^−5^ (95% HPD 8.2 × 10^−6^–2.2 × 10^−5^) substitutions/site/day (example reconstruction in Fig. 4B and C; highlighter plot in Fig. S4B) and a mean root date of May 1992 (95% HPD August 1989–October 1994), 3 years prior to diagnosis (Table S2). Again, HIV sequences sampled on ART (both proviral and HIV RNA) interspersed throughout the tree (Fig. 4B).

We next wished to “date” these sequences. In a prior study of a much smaller subgenomic data set from this participant, in which we only inferred one tree, we had fit separate linear models for the pre- and dual-ART periods because fitting a single model yielded a biologically implausible root date (13). On the present much larger data set where we inferred multiple trees, we explored dual regression, segmented regression, and single regression models and found that the latter yielded passing models for all trees, high likelihood scores, and biologically plausible date estimates with the narrowest 95% HPD intervals (data not shown). We therefore fit a single linear regression to BC-002’s data set. In doing so, the oldest sequence in the data set, a defective provirus, was estimated to have integrated in the first year of infection, making it nearly 21 years old at the time of sampling (Fig. 4D). Nevertheless, ~50% of sampled proviruses were estimated to have integrated in the 2.25 years prior to ART.

Like BC-001, all of BC-002’s oldest proviruses (those dating to before the initiation of dual ART) were defective. By contrast, the single intact provirus was estimated to have integrated in 2004, though many defective proviruses also dated to around this time. Notably, the HIV RNA sequences recovered during the persistent on-ART viremia period were genetically diverse and included sequences dating as far back as 1995. In fact, the sequences that emerged in plasma during this 2013 event were on average older than sampled proviruses (*P* = 0.027; Fig. 4D), an observation that remained consistent when we subsampled sequences from the proviral pool to the same depth as the on-ART viremia sequences (Fig. S5A). Four of the on-ART viremia sequences, one of which was recovered four times, mapped to a single subclade near the top of the tree, where the most closely related sequence was a provirus whose sole defect was a premature stop codon in Vif (Fig. 4B). This further supports the notion that defective proviruses can contribute to low-level viremia on ART (52), though we cannot rule out an unsampled intact provirus as the origin. Proviruses with large deletions were slightly older than those with Ψ defects (*P* = 0.043) though otherwise no significant age differences were found between defective proviral types (Fig. 4D).

#### Participant BC-003

**Fig 5:**
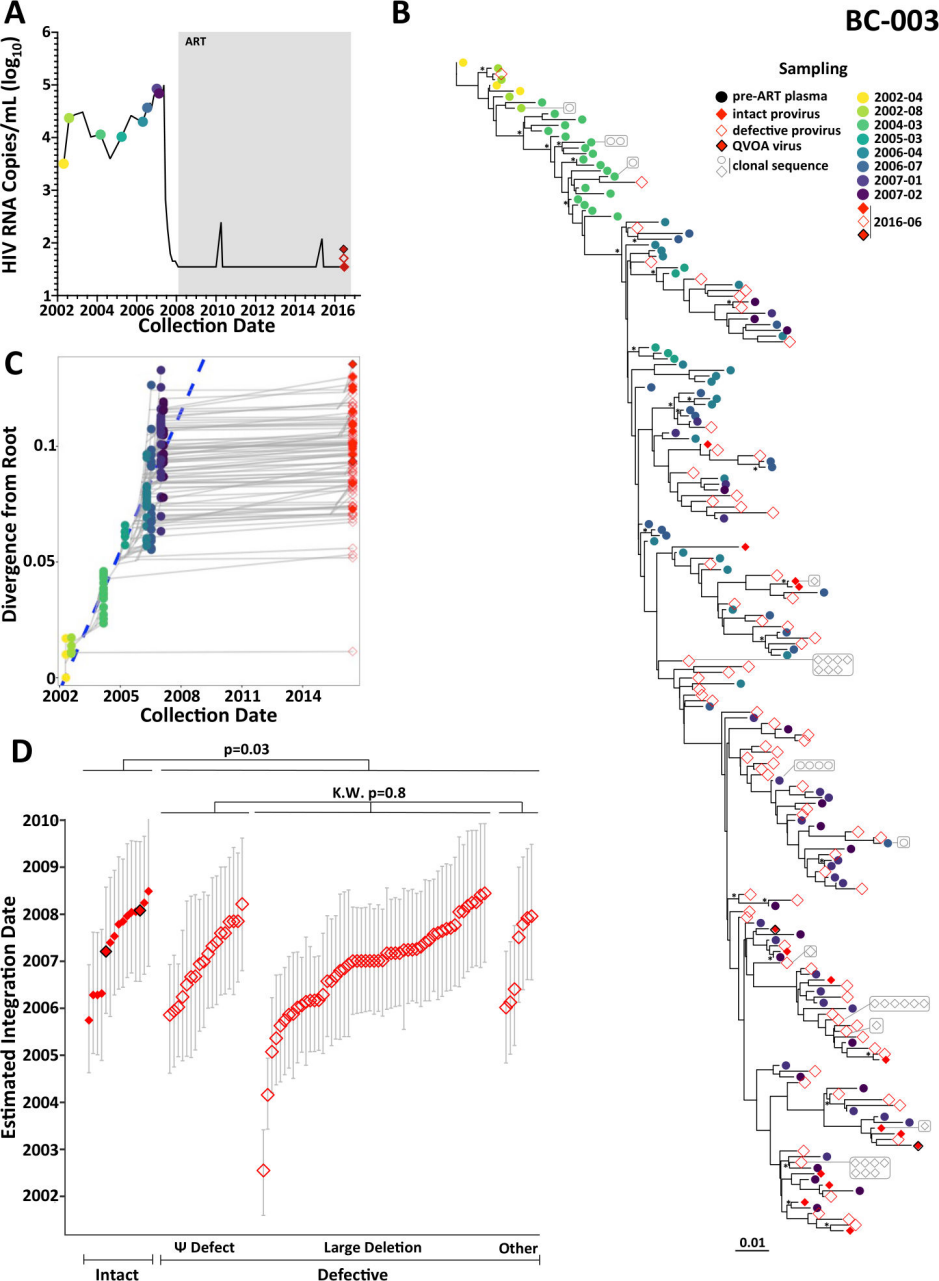
Within-host HIV evolutionary reconstruction and integration date inference of on-ART sequences for participant BC-003. The legend is the same as Fig. 3, except for the following modifications. For participant BC-003, only intact proviruses (solid red diamonds), defective proviruses (open red diamonds) and viral outgrowth (QVOA) sequences (solid red diamonds with a black outline) were dated.

Participant BC-003 was diagnosed with HIV in 2002. HIV was suppressed on ART in October 2007 and largely maintained for ~9 years, except for isolated low-level viremia (<250 HIV RNA copies/mL) in April 2010 and June 2015, from which HIV amplification was unsuccessful (Fig. 5A). We isolated 122 plasma HIV RNA *nef* sequences from eight pre-ART time points spanning 4.75 years, along with 733 proviruses on ART, of which 115 (16%) were *nef*-intact (Fig. 5A, Table 1). We also isolated one full and one partial HIV RNA genome from limiting-dilution QVOA performed on the same sample as the proviral isolation. All 4,500 within-host phylogenies showed strong molecular clock signal (Table S2), yielding a mean pre-ART *nef* evolutionary rate of 6.8 × 10^−5^ (95% HPD 4 × 10^−5^–1 × 10^−4^) substitutions/site/day and a mean root date of December 2001 (95% HPD February 2001–August 2002), which was only a few months prior to diagnosis (example reconstruction in Fig. 5B and C; highlighter plot in Fig. S4C). Overall, BC-003’s proviral pool was markedly skewed in age: all but two proviruses dated to the 2.75 years prior to ART (Fig. 5C and D). Nevertheless, intact proviruses and QVOA outgrowth viruses were on average significantly younger than defective proviruses (*P* = 0.03; Fig. 5D; subsampling validation in Fig. S5B). Though defective proviral types did not overall differ in age (Kruskal-Wallis *P* = 0.8), the oldest proviruses all harbored large deletions.

#### Participant BC-004

**Fig 6:**
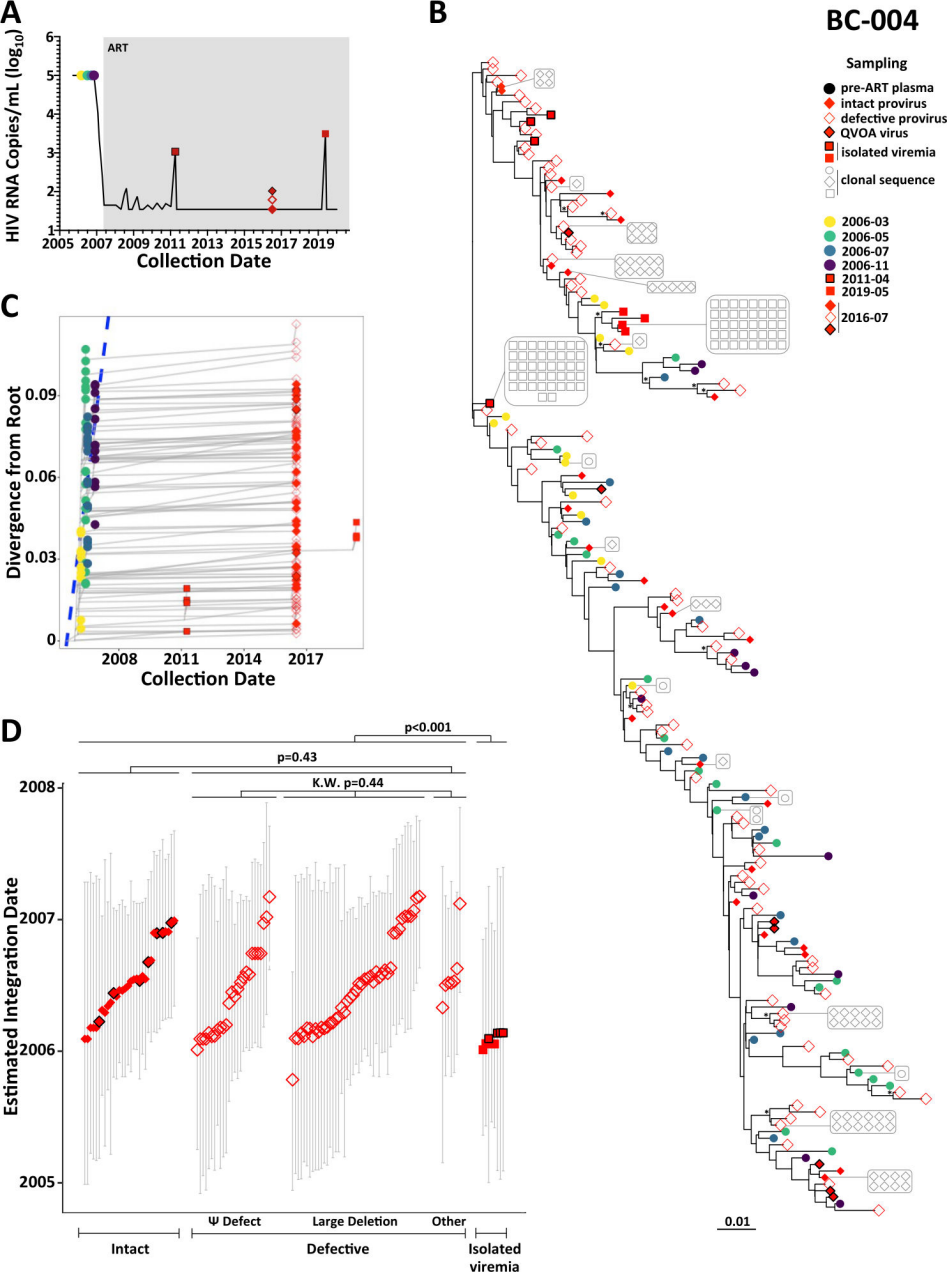
Within-host HIV evolutionary reconstruction and integration date inference of on-ART sequences for participant BC-004. The legend is the same as Fig. 3, except for the following modifications. For participant BC-004, intact proviruses (solid red diamonds), defective proviruses (open red diamonds), viral outgrowth (QVOA) sequences (solid red diamonds with a black outline) and plasma sequences from two instances of isolated viremia on ART were dated. For the latter, unique symbols are used to differentiate the 2011 (solid red square with a black outline) and 2019 (solid red square) isolated viremia events.

Participant BC-004 initiated ART approximately 2 years following diagnosis, which was much earlier than the other participants (Fig. 6A). Intermittent low-level viremia occurred in the first 4 years of ART, but suppression was maintained thereafter except for an isolated measurement of 3,120 copies/mL in 2019. There was no documented ART interruption at this time, and antiretroviral resistance genotyping predicted that all drugs retained full activity. We isolated 65 plasma HIV RNA *nef* sequences from four pre-ART time points spanning 8 months, along with 440 proviral genomes (165; 38% *nef*-intact), and seven unique HIV RNA genomes from QVOA in July 2016 (Fig. 6A; Table 1). We also isolated 46 (4 unique) and 44 (4 unique) plasma HIV RNA *nef* sequences from the on-ART viremia in 2011 and 2019, respectively. Of the 6,000 rooted within-host phylogenies, only 1,622 (27%) had sufficient molecular clock signal to pass quality control (Table S2), which is not surprising given that early ART initiation limits within-host HIV evolution (63–67). The passing trees yielded a mean pre-ART *nef* evolutionary rate of 1 × 10^−4^ (95% HPD 4.7 × 10^−5^–1.6 × 10^−4^) substitutions/site/day (example reconstruction in Fig. 6B and C; highlighter plot in Fig. S4D) and a mean root date of April 2005 (95% HPD October 2004–September 2005). This, combined with the limited viral diversity in this individual (Fig. S4D), is consistent with HIV diagnosis during early infection.

Due to the inherent uncertainty in phylogenies inferred from limited-diversity data sets, estimated integration dates for this participant have wide 95% HPD intervals and should be cautiously interpreted. Overall, BC-004’s intact and defective proviruses did not differ in terms of age (*P* = 0.43), nor did defective proviruses differ in age based on defect type (*P* = 0.44) (Fig. 6D). Nevertheless, the oldest provirus, estimated to have integrated in October 2005, 6 months following the estimated transmission date, was defective due to a large deletion (Fig. 6D). Moreover, sequences isolated from the on-ART viremia in 2011 and 2019 exclusively dated to early infection (January/February 2006) and, in fact, were on average older than sampled proviruses (*P* < 0.001) (Fig. 6D; subsampling validation in Fig. S5C). Both the 2011 and 2019 viremia events featured a dominant sequence (each isolated >40 times) plus three closely related singletons. All but one of these sequences fell within a single subclade (Fig. 6B, near top of tree), where the most closely related provirus sequence was defective.

#### Participant BC-021

**Fig 7:**
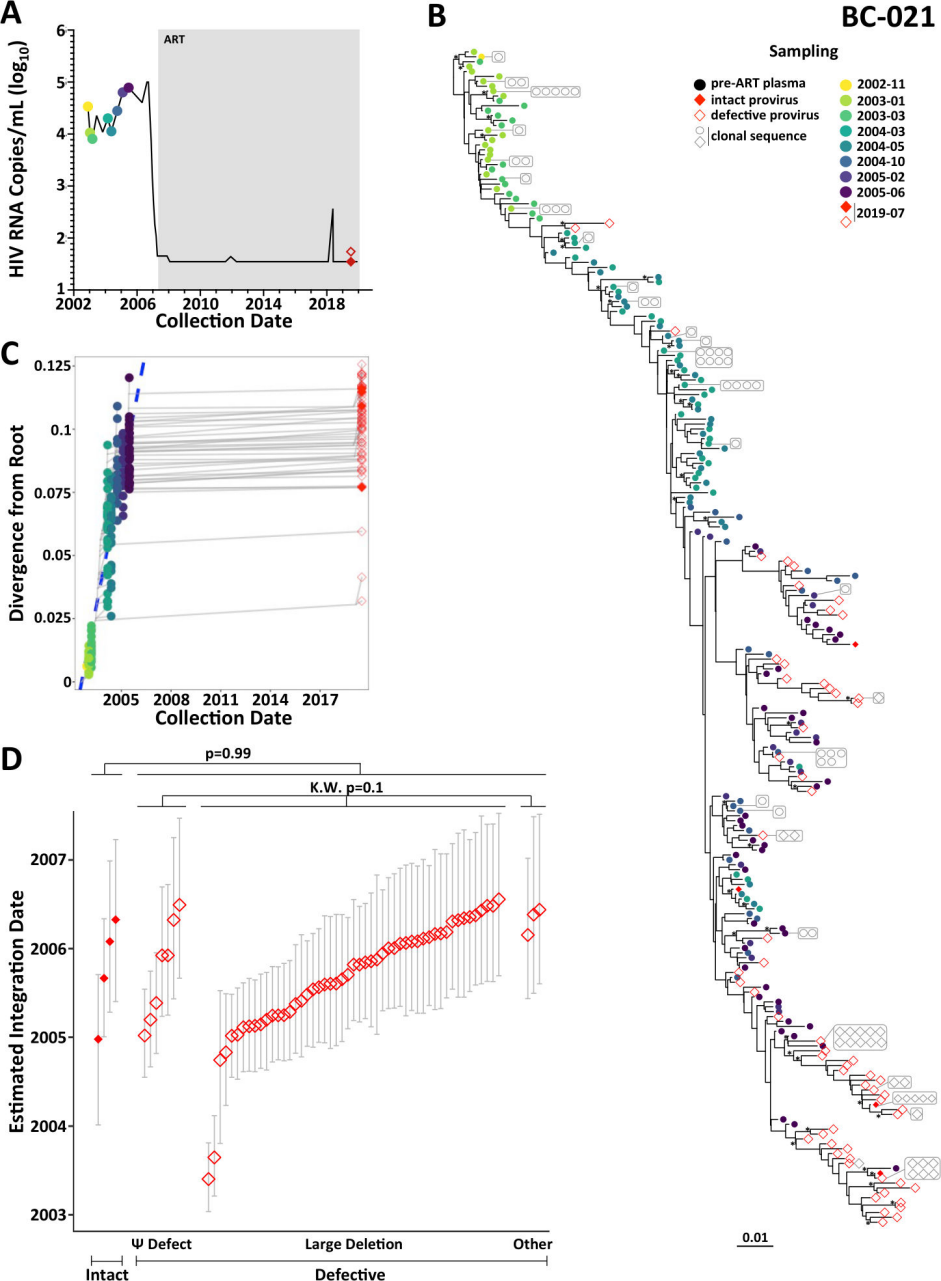
Within-host HIV evolutionary reconstruction and integration date inference of on-ART sequences for participant BC-021. The legend is the same as Fig. 3, except that for participant BC-021, only intact proviruses (solid red diamonds) and defective proviruses (open red diamonds) were dated.

Participant BC-021 was diagnosed with HIV in November 2002 and achieved viral suppression on ART in April 2007 (Fig. 7A). Suppression was largely maintained except for low-level viremia to 367 HIV RNA copies/mL in May 2018 from which sequence isolation was unsuccessful. We isolated 221 plasma HIV RNA *nef* sequences from eight pre-ART time points spanning 2.5 years and 386 proviruses on ART in July 2019, of which 93 (24%) were *nef*-intact (Fig. 7A; Table 1). All 3,000 rooted phylogenies demonstrated strong molecular clock signal (Table S2), yielding a mean pre-ART *nef* evolutionary rate of 8.4 × 10^−5^ (95% HPD 5.1 × 10^−5^ −1.2 × 10^−4^) substitutions/site/day (example reconstruction in Fig. 7B and C; highlighter plot in Fig. S4E) and a mean root date of April 2002 (95% HPD December 2001–August 2002), 7 months prior to diagnosis. BC-021’s on-ART proviral pool was markedly skewed in age, with all but five unique sequences dating to the 2.5 years prior to ART (Fig. 7D). Though intact and defective proviruses did not differ in age (*P* = 0.99), the only two proviruses originating from earlier in infection, both of which dated to 2003, 16 years prior to sampling, harbored large deletions (*P* = 0.1 for comparison with other defective proviral types, Fig. 7D).

#### Participant BC-027

**Fig 8:**
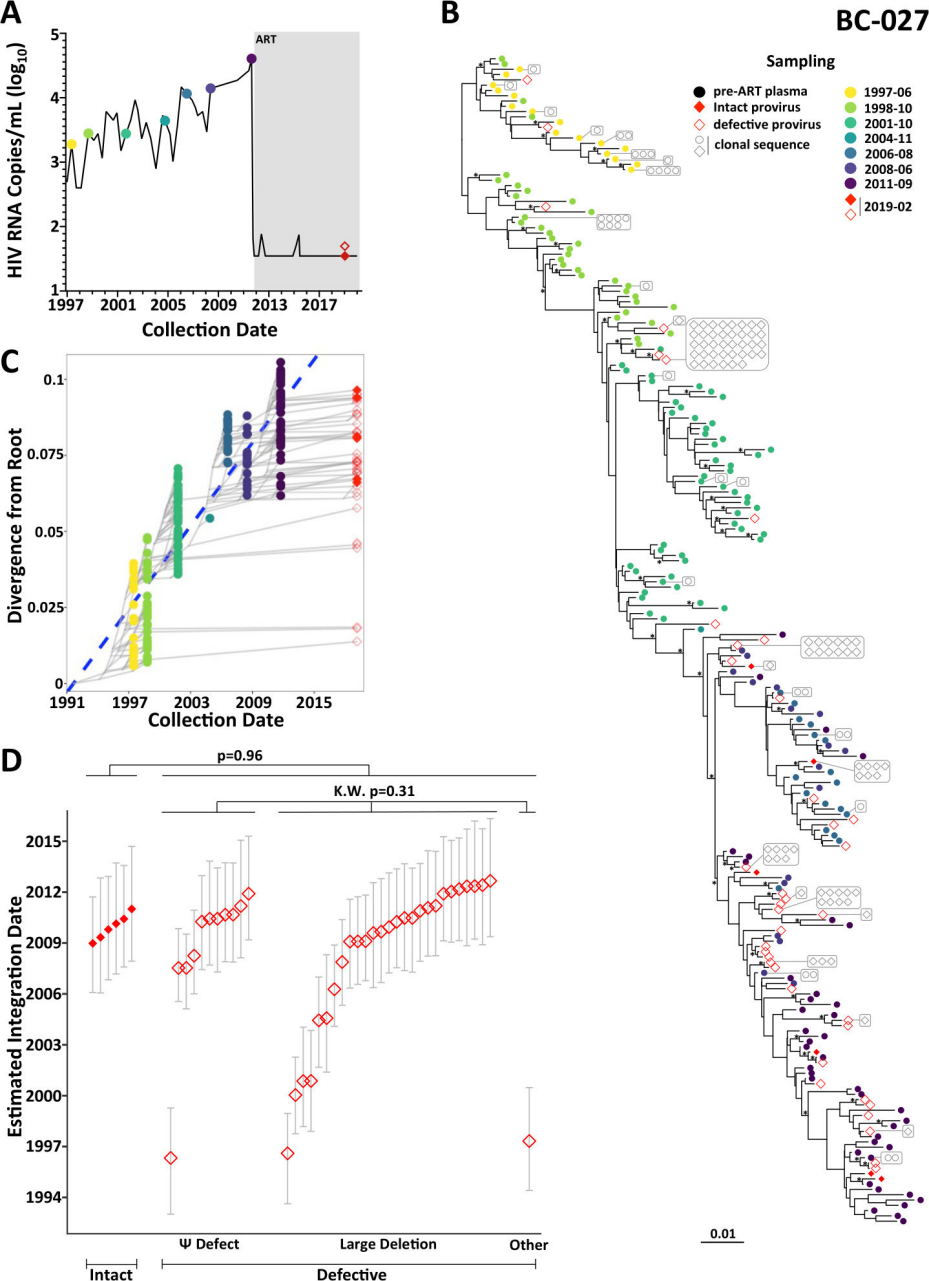
Within-host HIV evolutionary reconstruction and integration date inference of on-ART sequences for participant BC-027. The legend is the same as Fig. 3, except that for participant BC-027, only intact proviruses (solid red diamonds) and defective proviruses (open red diamonds) were dated.

Participant BC-027 was diagnosed with HIV in 1985. Viral load testing, established in British Columbia in 1996, revealed that the participant exhibited relative natural viremia control around this time. The participant initiated ART in November 2011 (Fig. 8A). Suppression was maintained except for two low-level viremia events <80 HIV RNA copies/mL from which sequence isolation was unsuccessful. We isolated 215 pre-ART plasma HIV RNA *nef* sequences from seven time points spanning a 14-year period, along with 195 proviruses on-ART in 2019, of which 127 (65%) were *nef*-intact (Fig. 8A, Table 1). All 1,500 within-host rooted phylogenies displayed strong molecular clock signal (Table S2), yielding a mean (95% HPD) pre-ART *nef* estimated evolutionary rate of 1.5 × 10^−5^ (9.1 × 10^−6^–2.1 × 10^−5^) substitutions/site/day (example reconstruction in Fig. 8B and C; highlighter plot in Fig. S4F). The mean root date was September 1992 (95% HPD January 1990–March 1995), indicating that we did not reconstruct back to the founder virus but rather to one of its descendants. Assuming that this root placement was correct, there was no difference in integration dates between intact and defective proviruses (*P* = 0.96), nor between different types of defective proviruses (Kruskal-Wallis *P* = 0.31) (Fig. 8D). Nevertheless, all of the oldest proviruses, the most long lived of which dated to 1996 (23 years prior to sampling) by this rooting method, were defective.

One potential concern is whether our primary rooting method correctly placed the root for this individual. To investigate this, we re-calculated all on-ART proviral integration dates after out-group rooting each of BC-027’s phylogenies. Out-group rooting produced tree statistics that were highly comparable to our original rooting method: namely, an *nef* evolutionary rate of 1.6 × 10^–5^ (9.5 × 10^–6^–2.2 × 10^–5^) substitutions/site/day (example reconstruction in Fig. S6B and C) and a mean root date of February 1991 (95% HPD September 1987–June 1994). These observations further supported the notion that no sequences dating to early infection remained in BC-027’s data set. Importantly, the proviral integration dates inferred using both rooting methods were essentially identical (Lin’s concordance correlation coefficient 0.986), except for one defective provirus that the primary method dated to 1996, but out-group rooting dated to late 1991 (Fig. S7). Also consistent with the primary analysis, out-group-rooted integration dates did not differ between intact and defective proviruses (*P* = 0.96) nor between defective provirus types (*P* = 0.31; Fig. S6D), and the oldest recovered proviruses were exclusively defective. Taken together, these results indicate that BC-027 was the only participant for whom no proviruses were recovered that dated to the very earliest years of infection. This observation is consistent with a similar evolutionary characterization of on-ART proviruses in an individual who initially controlled viremia, but later lost this control and initiated ART (17).

#### Cross-participant comparison

**Fig 9:**
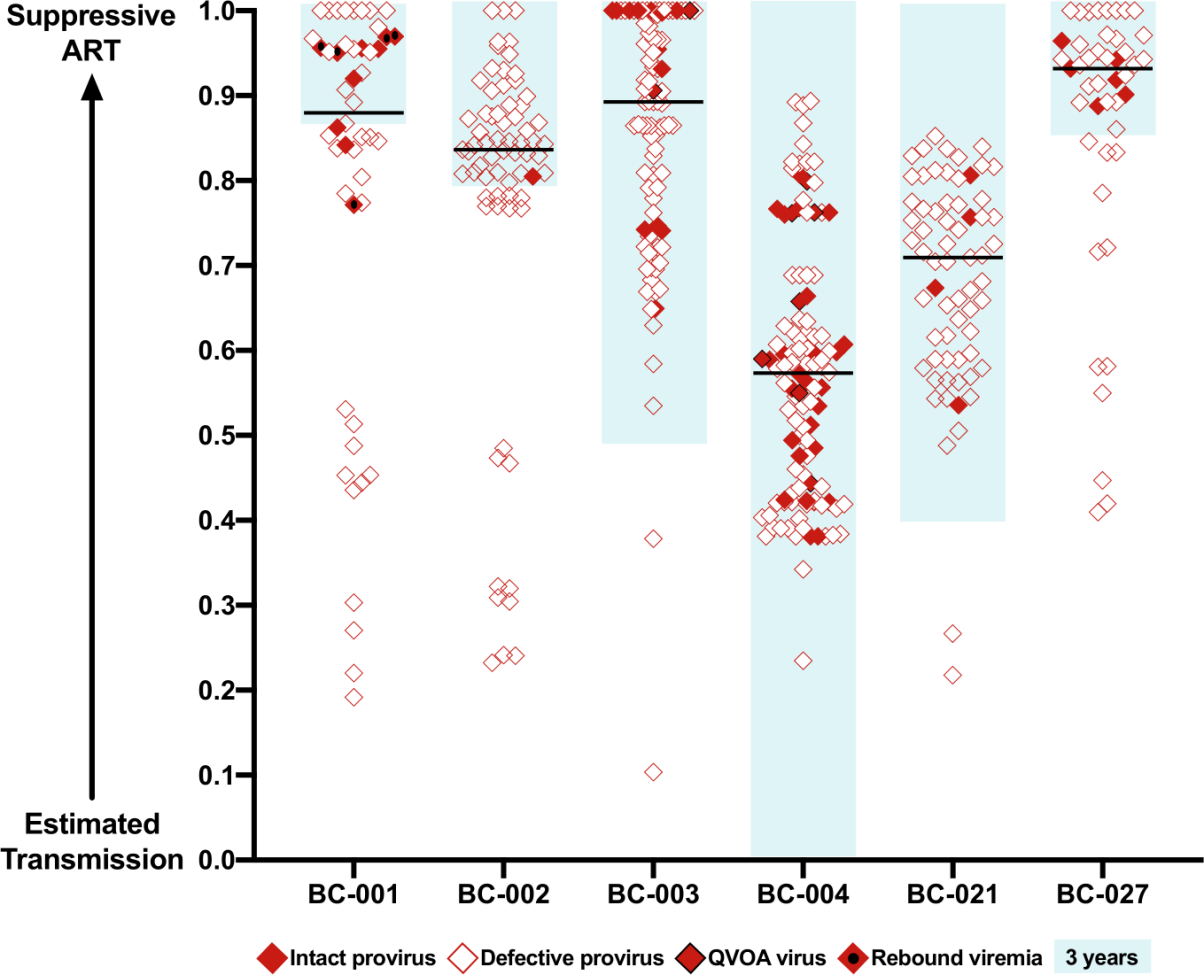
Scaled integration timings of intact and defective sequences for all participants. Mean estimated integration timings of intact sequences (proviral: solid red diamonds; QVOA: solid red diamonds with a black outline; rebound viremia: solid red diamonds with a black dot) and defective sequences (open red diamonds), where these timings are presented on a scale between estimated HIV transmission (obtained from clinical records, participant-reported date, or the mean root date of within-host phylogenies, whichever was earliest; see Table 1) and ART suppression (not initiation). For participant BC-001, the date of durable ART suppression, that occurred after the rebound, was used. Note that low-level/isolated viremia sequences are not plotted, as it is not known whether these are intact or defective. Sequences with integration dates after ART suppression were plotted at 1.0. The black horizontal line denotes the median. The blue shaded box marks the 3 years prior to ART suppression in each participant.

In order to facilitate cross-participant comparisons of on-ART proviral integration dates, we expressed these dates on a scale between estimated infection and first durable ART suppression (Fig. 9). These integration dates are markedly skewed towards the 3 years prior to ART-mediated suppression (Fig. 9, blue boxes) in all participants except BC-004, who was the only participant who initiated ART within this timeframe. It is also clear that the oldest sequences recovered are defective proviruses in all cases (note that low-level/isolated viremia sequences are not represented on this plot, as genomic integrity could not be established).

### Clones do not differ from unique sequences in terms of age

We investigated whether clonal sequences differed from unique ones in terms of their overall age distribution. Here, as in previous analyses, members of a clonal set were collapsed into a single data point for analysis. Also, sequences from plasma viral rebound and on-ART viremia were not considered, as only *nef* was sequenced and therefore clonality could not be established. We observed no significant differences in age distribution between clonal and unique sequences in any participant (Fig. S8, all *P* > 0.1). This remained true when sequences were stratified by genomic integrity (all *P* > 0.12).

## DISCUSSION

In this study, we used phylogenetic methods to explore the relationship between proviral genomic integrity and longevity, as well as the within-host origins of plasma viremia. As expected (49), given that proviruses were sampled after an average of more than 9 years on ART, only 4% were intact, with some participants (e.g., BC-002) having as few as 0.4% intact. Proviruses with large deletions typically dominated, where, consistent with previous studies (38, 49), we identified shared HIV genomic breakpoints that illuminate how such deletions reproducibly occur, both within and across individuals.

Our observation that intact and Ψ-defective proviruses were nearly three times more likely to be clonal, while hypermutated proviruses were twofold less likely, extends our understanding of clonal expansion in reservoir maintenance (21, 31, 38–42, 44, 45). Some of these differences likely reflect cellular distribution (57), as intact and Ψ-defective proviruses tend to be enriched in effector memory CD4^+^ T-cells (39, 44, 57, 68), which have proliferated (69), while hypermutated proviruses tend to be enriched in naive CD4^+^ T-cells (39, 44, 57, 68, 70), which have not substantially proliferated (69). These results are also consistent with intact and Ψ-defective proviruses being more dependent on clonal expansion for survival because they are at higher risk of elimination by cytopathic effects or immune responses after reactivation (at least some Ψ-defective proviruses can produce HIV proteins (71) and even virions (52)). It is important to note, though, that the mechanism of clonal expansion—whether antigen-, homeostatic-, or integration-site driven (21, 30–47)—cannot be deduced from sequences alone. As not all members of a clone will reactivate upon stimulation (72, 73), expansion enhances the likelihood that at least some members will persist (74). In contrast, grossly defective proviruses rely less on clonal expansion for survival, as their risk of elimination is inherently lower due to limited or no viral antigen presentation (21, 53).

Also consistent with prior studies (14–16, 29), participants’ on-ART proviral pools ranged from modestly (e.g., BC-001) to substantially (e.g., BC-003 and BC-021) skewed toward viral variants archived in the years immediately preceding ART. This is consistent with continual reservoir seeding—and turnover—during untreated infection, such that, if ART is not initiated until chronic infection, many ancestral within-host lineages will have already been eliminated by this time (14, 17, 18). Nevertheless, and consistent with prior studies that sampled subgenomic proviral sequences on ART (13–16, 28), we recovered at least one sequence dating to early infection in all but one participant (BC-027). The oldest recovered proviruses were exclusively defective, usually due to large deletions, whereas intact proviruses dated to nearer ART initiation, indicating that intact proviruses have shorter lifespans than those with gross defects. This is consistent with the more rapid decay of intact proviruses during ART (21–23, 25, 26), and likely during untreated infection as well (14, 17, 18), though the latter has not been explicitly demonstrated.

The observation that the oldest proviruses persisting during long-term ART are exclusively defective also helps resolve a discordance in the literature. It explains why studies that recovered subgenomic proviral sequences on ART (which are largely defective) routinely recovered proviruses dating to early infection (13–16, 28), whereas the study that exclusively dated *ex vivo* viral outgrowth sequences sampled on ART yielded hardly any sequences dating to this time (29). The latter study nevertheless did find a handful of intact viruses dating to early infection, whereas we found none (except in BC-004, for whom all proviruses dated to the short period between infection and ART). This may be because both the time to ART initiation (except for BC-004) and the time on ART were substantially longer in the present study, allowing more time for intact proviruses to be eliminated *in vivo*. Nevertheless, we acknowledge the possibility that older intact proviruses do persist *in vivo*, but that we did not sample to a sufficient depth to identify any in the present study. Taken together with existing data (40, 41, 44), our findings nonetheless indicate that intact proviruses are at a survival disadvantage compared to their grossly defective counterparts and are thereby more dependent on clonal expansion for persistence.

Our observations also suggest that low-level/isolated viremia on ART can have distinct within-host origins from rebound viremia. Though the sequence recovered from BC-001’s low-level viremia dated to the year before ART, the sequences recovered from BC-002 and BC-004 were heterogeneous in terms of age and genetics. This further suggests that some older proviruses, including those with defects, can reactivate to produce viremia (though again we cannot rule out that viremia arose from older, intact proviruses that remained unsampled). The existence of genetically defective virions is supported by their presence during untreated infection (though recently infected, rather than reservoir cells, would be the likely source during that time) (75), as well as during persistent low-level viremia on ART (52). Indeed, the recent finding that some proviruses with 5′ leader defects produce virions lacking envelope on their surface provides a mechanism whereby they—and the cells producing them—could evade antibody responses and thus persist long term (52). By contrast, HIV sequences rebounding to high levels in plasma after ART interruption dated to the years just prior to ART (BC-001), consistent with an origin from intact proviruses from that period. Of note, proviruses highly genetically similar to those rebound viruses were still present 8 years after ART initiation. While this is consistent with the rebound event having re-seeded the reservoir to some extent, it is important to note that the proviruses that persisted long-term after the rebound may no longer fully reflect the original population, as any long-lived proviruses would have endured years of selection—not on the viral genome, but on features such as integration site and clonal expansion—in the years after the rebound event.

Our observation that intact proviruses (and rebound viremia) exclusively dated to the later years of untreated infection has implications for immune-based cure strategies because sequences from this period will have substantially adapted to within-host selective pressures (Fig. S4 illustrates the selective sweeps that typify this process (67, 76)). Indeed, human leukocyte antigen (HLA) class I-restricted escape mutations were apparent in the five participants who initiated ART in advanced chronic infection; these included the C*06:02-restricted Nef-125H adaptation (77) at position 6 of the C*06-restricted-YT9 epitope (Nef 120–128) (78, 79) in BC-001, the C*07:01-restricted Nef-105Q adaptation (77) at position 1 of the C*07-restricted KY11 epitope (Nef 120–128) (77) in BC-003, the B*35:01-restricted Nef-81F adaptation (77) at the C-terminus of the B*35:01-restricted VY8 epitope (Nef 74–81) (80) in BC-027, and others. Studies of rebound HIV from larger numbers of individuals in a within-host evolutionary context will help establish whether rebound sequences are on average even more adapted to host immune responses than the overall intact proviral pool. If so, this would be consistent with the notion that rebound is a selective process, where the viruses that first appear in plasma at high levels are not necessarily those that reactivated first, but rather those that host immune responses, particularly antibodies, subsequently fail to control (81, 82). This may also explain why *in vivo* rebound and *in vitro* outgrowth HIV do not always match (83).

An elegant study recently undertaken in a simian immunodeficiency virus (SIV) model (84) reported an observation that may at first seem discordant with our observation that HIV reservoirs (defined as intact, replication-competent proviruses) are dominated by genetically “younger” and more immune-adapted sequences. But, these observations can potentially be reconciled. As previously reported for HIV (9–16, 21–27), the SIV study confirmed that the proviral pool at ART initiation retained SIV sequences that had integrated throughout untreated infection, consistent with continual reservoir seeding, but that thereafter, intact and defective SIV genomes decayed with markedly different kinetics. The study further demonstrated that, early after ART initiation, proviruses with high burdens of antibody escape mutations (i.e., younger proviruses that integrated near ART initiation) were preferentially lost, such that older proviruses with fewer escape mutations became relatively more prominent over time on ART. A similar shift toward older sequences was also observed in four human participants for whom subgenomic proviral sequences were sampled twice during ART (15). These observations, however, do not necessarily contradict ours. First, while both the SIV and human study found evidence that recently infected cells are preferentially eliminated after ART initiation, proviruses of widely varying ages persisted throughout follow-up. More importantly, because both prior studies employed subgenomic sequencing, it is not possible to establish whether the age distributions of intact and defective proviruses differed after long-term ART. The timescales of the non-human primate and present studies also differed markedly. While the animals initiated ART within 48 weeks of infection, the present participants initiated ART after 9 years, leaving open the possibility that the genetic (and age) distributions of intact and defective proviruses already differed at ART initiation in the two studies, thereby influencing their subsequent distribution during ART. Similarly, on-ART follow-up was shorter for the non-human primate study (4 years) versus a median of 8.9 years in the present one. Further reservoir evolutionary dynamics studies that distinguish intact from defective proviruses, ideally measured longitudinally on ART, will further help reconcile these observations.

Our study has some limitations. Despite extensive sampling, we recovered fewer intact proviruses than predicted based on the IPDA, reflecting the inefficiency of long-range PCR (56). It is, therefore, indeed possible that older intact proviruses persist during long-term ART, but at too low frequencies to be identified at the present sampling depth. It is also worth noting that not all genome-intact proviruses are replication competent, as factors such as integration site can influence reactivation capacity (35, 37). Conversely, it is possible that a minority of proviruses that we classified as defective (e.g., those with defects in an accessory protein) can replicate. Since we did not determine the integration site, we cannot definitively state that identical sequences are clonal, though the likelihood is high as a previous study demonstrated that proviruses with 100% sequence identity also share integration sites (85). We also acknowledge that sequences isolated only once may still be part of a clonal set (86). Within-host HIV sequences of interest were dated using a model that assumes a strict molecular clock, which may not be ideal over long time frames (87). Nevertheless, the observation that the oldest proviruses are defective is apparent even without fitting a model, as these were always the closest to the root of the phylogeny. Moreover, when we applied an alternative dating method to the participants with the longest untreated infection times, which allows variable evolutionary rates over the tree’s edges (88, 89), we obtained integration dates that correlated strongly with the original results (all Spearman’s *r* > 0.86, *P* < 0.0001; not shown). As we could only date *nef*-intact proviruses, we cannot rule out that *nef*-defective proviruses have different age distributions, which is possible as *nef* may promote proviral longevity (57). Nevertheless, studies that have used *gag* and *env* for dating (14–16, 29) have produced similar proviral age distributions, suggesting that our results are not overly biased. There are also no ideal solutions to this, as the abundance of large deletions means that no single HIV region can be used to date all proviruses phylogenetically, nor can hypermutated sequences be dated using such approaches. Lastly, it is important to note that our use of “old” and “young” to describe sequences dating to earlier infection versus near ART initiation, respectively, is appropriate only for individuals who did not initiate ART until well into chronic infection, such as those studied here.

Despite these limitations, our study is the first to compare the ages of defective and intact proviruses on ART, along with reservoir-origin HIV RNA, in the context of within-host HIV evolutionary history. As participants were sampled after a median of 8.9 years on ART, recovered sequences represent truly long-lived proviruses. We addressed within-host phylogenetic reconstruction uncertainty by inferring 1,500–6,000 trees per participant. Rather than using clustering approaches, which can only date sequences of interest to the specific time points when plasma HIV RNA was sampled pre-ART (29, 88), our regression approach allowed us to date sequences to before (or after) pre-ART plasma sampling.

In summary, in this cohort of individuals who did not initiate ART until chronic infection, the oldest proviruses persisting during long-term ART were exclusively genetically defective. In contrast, intact proviruses and rebound HIV RNA dated nearer to ART initiation and were enriched in mutations consistent with accumulated adaptation to host pressures. Intact proviruses were also more likely to be clonal. This indicates that genome-intact proviruses have shorter average lifespans than grossly defective ones, likely due to increased risk of viral reactivation, antigen production, and elimination (50, 53, 71), and therefore that they are more dependent on clonal expansion for survival than their defective counterparts. By contrast, on-ART viremia sometimes belonged to very old, and possibly defective, within-host lineages (52), underscoring the need to better understand the biological and clinical implications of these long-lived defective lineages. Overall, our results provide further evidence that cure strategies will need to eliminate an intact viral reservoir that is clonally enriched, genetically younger, and thus more adapted to its host.

## MATERIALS AND METHODS

### Participants and sampling

We recruited six participants living with HIV. All were male and had initiated triple ART, a median of 11 (range 2.2–26.5) years after estimated HIV infection (Table 1). At the time of peripheral blood mononuclear cell (PBMC) sampling on ART, plasma viral loads had been largely suppressed for a median of 8.9 (range 7.2–12.2) years. A median of 8 (range 4–17) pre-ART longitudinal plasma samples per participant were used to reconstruct within-host HIV evolutionary histories. We also studied plasma from a viremia rebound event following ART interruption in one participant (BC-001) and plasma from on-ART low-level and/or isolated viremia events in three participants (BC-001, BC-002, and BC-004). HLA Class I typing was performed on DNA extracted from whole blood or CD4^+^ T-cells, using a sequence-based method (90).

### Amplification and sequencing of plasma HIV RNA

HIV RNA *nef* sequences were isolated from longitudinal pre-ART plasma samples, rebound viremia, and on-ART viremia as follows. Total nucleic acids were extracted from 500 mL of plasma on the NucliSENS EasyMag (bioMerieux) and subjected to DNase treatment if the original plasma viral load was <250 HIV RNA copies/mL. cDNA, generated using HIV-specific primers, was endpoint-diluted such that subsequent nested PCR reactions yielded no more than 30% positive amplicons, as previously described (13, 17). Amplicons were sequenced using a 3130xl or 3730xl Automated DNA Sequencer (Applied Biosystems), and chromatograms were analyzed using Sequencher (v.5.0, Gene Codes). Sequences with nucleotide mixtures, hypermutation, or suspected within-host recombination (identified using RDP4 [91]) were removed prior to phylogenetic inference. Of note, and consistent with a recent report (75), we did occasionally observe defective plasma HIV RNA *nef* sequences (e.g., *nef* sequences with an internal stop codon were isolated at various time points from participant BC-021). HIV drug resistance genotyping was performed on select on-ART viremia samples using standard approaches (92), and interpretations were performed using the Stanford HIV drug resistance database (93).

### Intact proviral DNA assay (IPDA)

Intact and total proviral DNA was quantified in CD4^+^ T-cells isolated by negative selection from on-ART PBMC using the IPDA (48, 94), as previously described (94), where XhoI restriction enzyme (New England Biolabs) was added to each ddPCR reaction to aid in droplet formation per the manufacturer’s recommendation. There are no XhoI cut sites between the two human (RPP30) target regions that are used to quantify the number of cellular genomes and determine the extent of DNA shearing in the extract, nor between the HIV IPDA target regions for any of the intact provirus sequences collected for this study. For participant BC-004, an autologous *env* probe (VIC-CCTTGGGTT**TC**TGGGA-MGBNFQ) was used, where the bold and underlined bases represent modifications from the published IPDA probe, which failed due to these polymorphisms (94).

### Near-full-length HIV proviral amplification and sequencing

Single-template, near full-length HIV proviral amplification was performed on genomic DNA extracted from CD4^+^ T-cells using Platinum Taq DNA Polymerase High Fidelity (Invitrogen), where IPDA-determined total proviral loads were used to dilute DNA such that no more than 30% of resulting nested PCR reactions yielded an amplicon (protocol described in reference 40). Amplicons were sequenced (Illumina MiSeq) and reads were *de novo* assembled using an in-house modification of the Iterative Virus Assembler (95) implemented in the custom software MiCall (http://github.com/cfe-lab/MiCall) to generate a consensus sequence. Each sequence was verified to have been sequenced end-to-end by locating at least part of the second round PCR primers at both 5′ and 3′ ends and to have a minimum read depth of ≥100 over all nucleotide positions. Sequences not meeting these criteria were discarded. We validated our pipeline by single-genome sequencing the provirus integrated within the J-Lat 9.2 cell line (96) in 146 independent replicates, as well as by sequencing a panel of nine in-house engineered pNL4-3 plasmids in which we had deleted large HIV genomic regions (97–100). The J-Lat validation yielded 25 consensus base errors out of a total 1,375,174 bases sequenced (i.e., an error rate of 1.8 × 10^−5^), while the plasmid validation correctly reconstructed the deletion breakpoints 100% of the time. The genomic integrity of sequenced proviruses was determined using an in-house modification of the open-source software HIV SeqinR (101), where an intact classification required all HIV reading frames, including accessory proteins, to be intact. Intactness classifications for all non-hypermutated proviruses longer than 8,000 bases were manually confirmed by checking each HIV gene for the presence of start and stop codons (where applicable), internal stop codons, hypermutation, or other defects, and checking for known defects in the packaging signal region (38). Sequences with 100% identity across the entire amplicon were considered identical and clonal.

### Sequence isolation from quantitative viral outgrowth assay (QVOA)

QVOA (102) was performed for four participants for whom sufficient biological material was available (BC-001, BC-002, BC-003, and BC-004), as previously described (94). Briefly, CD4^+^ T-cells were isolated from PBMCs by negative selection and plated in serial dilution at either four or six concentrations (12 replicate wells/concentration, 24-well plates, range 18.6–26.8 million CD4^+^ T-cells total). CD4^+^ T-cells were stimulated with phytohemagglutinin (PHA; 2 µg/mL) and irradiated allogeneic HIV-negative PBMCs were added to further induce viral reactivation. MOLT-4/CCR5 cells were added at 24 hours post-stimulation as targets for viral infection. Culture medium (RPMI 1640 + 10% fetal bovine serum + 1% Pen/Strep + 50 U/mL IL-2 + 10 ng/mL IL-15) was changed every 3 days, and p24 ELISA was run on day 14 to identify virus-positive wells. Culture supernatants from virus-positive wells were frozen at −80°C until analysis. Only two participants, BC-003 and BC-004, yielded viral outgrowth. For these, near-full length HIV RNA genomes were RT-PCR amplified from culture supernatants from virus-positive wells where outgrowth was likely to be clonal (<30% positive wells at the specific dilution) in five overlapping amplicons, sequenced on an Illumina MiSeq, and assembled as described above.

### Within-host HIV evolutionary reconstruction and phylogenetic dating

Within-host phylogenies were inferred from *nef* sequence alignments comprising all unique plasma, proviral, and QVOA sequences collected per participant. To mitigate uncertainty in within-host HIV phylogenetic reconstruction, we inferred a median of 2,250 (range 1,500–6,000) phylogenies per participant using Bayesian approaches to allow us to flexibly capture the diversity unique to each within-host data set (Table S2). To do this, the best-fitting substitution model for each data set was first determined using jModelTest version 2.1.10 (103). The best fit for BC-002 was the HKY + I + G (Hasegawa-Kishono-Yano model with invariable site plus gamma distribution with four rate categories); for all others, it was the GTR + I + G (General Time Reversible model with invariable site plus gamma distribution with four rate categories). Markov Chain Monte Carlo (MCMC) methods were then used to infer a random sample of phylogenies for each participant. Phylogenetic inference was agnostic to sequence sampling time. Two parallel runs with MCMC chains of five million generations each, sampled every 10,000 generations, were performed in MrBayes version 3.2.5 (104) without enforcing a molecular clock, using the best-fitting nucleotide substitution model and model-specific or default priors, where appropriate. Between 10 and 40 million MCMC generations were run for each participant. Convergence was assessed by ensuring the deviation of split frequencies was <0.03, the effective sample size of all parameters was ≥200, and through visual inspection of parameter traces in Tracer version 1.7.2 (105). A split frequency of <0.03, rather than the more stringent <0.01, was chosen because our goal was to generate a random sample of phylogenies from the posterior over which we could then integrate our analyses, not to achieve a single well-resolved tree topology (which would require a more stringent cut-off). The first 25% of generations were discarded as burn-in. Node support values are derived from Bayesian posterior probabilities from the consensus tree. Bayesian within-host phylogenetic inference details are summarized in Table S2.

Integration dates of on-ART sequences were inferred using a phylogenetic approach (13). First, each participant’s phylogenies were rooted at the estimated most recent common ancestor by identifying the location that maximized the correlation between the root-to-tip distance and the sampling date of the pre-ART plasma HIV RNA sequences, as within-host sequence divergence from the transmitted/founder virus increases over time (87, 106, 107). Linear regression was then used to relate the sampling dates of the pre-ART plasma HIV RNA sequences and their root-to-tip divergence, where the slope of the regression represents the average within-host *nef* evolutionary rate, and the *x*-intercept represents the root date. We assessed model fit by comparing the Akaike information criterion (AIC) of the model to that of the null model (a zero slope), where a ΔAIC ≥ 10 was required to pass quality control. A median of 1,561 (IQR 1,500–3,375) phylogenies per participant yielded passing linear models. These were used to convert the root-to-tip distances of on-ART sequences to their estimated integration dates, which were then averaged across all passing models per participant. Highest posterior density intervals were computed using the R package HDInterval (version 0.2.2). Participant root date estimates are summarized in Table S2.

For participant BC-027, the same approach was applied to phylogenies that were out-group-rooted using the evolutionary placement algorithm in RAxML, implemented in a custom script in R (108). Here, the most closely related *nef* sequence from participant BC-021 (Fig. S1) was used as the out-group. After pruning off the branch leading to the out-group, on-ART sequences were dated using the same approach as described above.

Between-host phylogenies (Fig. S1 and S2) were inferred using *gag* and *nef* sequences under a maximum likelihood model. Briefly, sequences were aligned in a codon-aware manner in MAFFT version 7.475 (109) and manually edited in AliView version 1.19 (110). Phylogenies were inferred with IQ-TREE 2 (111) following automated model selection using ModelFinder (112) with an AIC selection criterion. Branch support values were derived from 1,000 bootstraps. Phylogenies were visualized using the R package ggtree (113).

### Statistical analyses

Unless otherwise indicated, statistics were computed using GraphPad Prism 8 or R version 4.2.1 implemented in RStudio v2021-01-06 or greater.

## Data Availability

Previously published sequences used in this study have the following accession numbers: MG822917, MG822918, MG822920–MG822961, MG822963–MG823015, MN600002–MN600010, MG823016, MG823017, MG823019–MG823143, MN600011–MN600053, MN600054–MN600175, and MN600183–MN600247. GenBank accession numbers for the HIV RNA *nef* sequences from pre-ART plasma and rebound and on-ART viremia collected in this study are OQ723958–OQ724483 and OQ750829–OQ750838. GenBank accession numbers for defective HIV proviral sequences are: OQ750839–OQ753083, while those for intact HIV proviruses and QVOA outgrowth viruses are OQ939945, OR454048–OR454052, OR466092–OR466114, OR483970–OR483984, OR483985–OR484031, OR502359–OR502366.

## SUPPLEMENTAL MATERIAL

Supplemental Figures mbio.02417-23-s0001.pdf Figures S1 to S8.

Table S1 mbio.02417-23-s0002.docx Participant reservoir composition and size.

Table S2 mbio.02417-23-s0003.docx Bayesian within-host phylogenetic inference details.

ASM does not own the copyrights to Supplemental Material that may be linked to, or accessed through, an article. The authors have granted ASM a non-exclusive, world-wide license to publish the Supplemental Material files. Please contact the corresponding author directly for reuse.
