# Supplementary material for: HIV reservoirs are dominated by genetically younger and clonally enriched proviruses
Source: mBio. 2023 Nov 16;14(6):e02417-23. doi: 10.1128/mbio.02417-23 (PMC10746175; doi:10.1128/mbio.02417-23)
Supplement: Supplemental Figures — Figures S1 to S8. [file mbio.02417-23-s0001.pdf]

Supplemental Figures for

HIV reservoirs are dominated by genetically younger and clonally enriched proviruses

Natalie N. Kinloch[1,2], Aniqa Shahid[1,2], Winnie Dong[2], Don Kirkby[2], Bradley R. Jones[2,3], Charlotte J. Beelen[2], Daniel MacMillan[2], Guinevere Q. Lee[4], Talia M. Mota[4], Hanwei Sudderuddin[2,5], Evan Barad[1,2], Marianne Harris[2,6], Chanson J. Brumme[2,7], R. Brad Jones[4], Mark A. Brockman[1,8], Jeffrey B. Joy[2,3,7], Zabrina L. Brumme[1,2]

[1]Faculty of Health Sciences, Simon Fraser University, Burnaby, BC
[2]British Columbia Centre for Excellence in HIV/AIDS, Vancouver, BC
[3]Bioinformatics Program, University of British Columbia, Vancouver, BC
[4]Infectious Diseases Division, Department of Medicine, Weill Cornell Medical College, New York, NY, USA
[5]Experimental Medicine Program, University of British Columbia, Vancouver, BC
[6]Department of Family Practice, Faculty of Medicine, University of British Columbia, Vancouver, BC
[7]Department of Medicine, University of British Columbia, Vancouver, BC
[8]Department of Molecular Biology and Biochemistry, Faculty of Science, Simon Fraser University, Burnaby BC

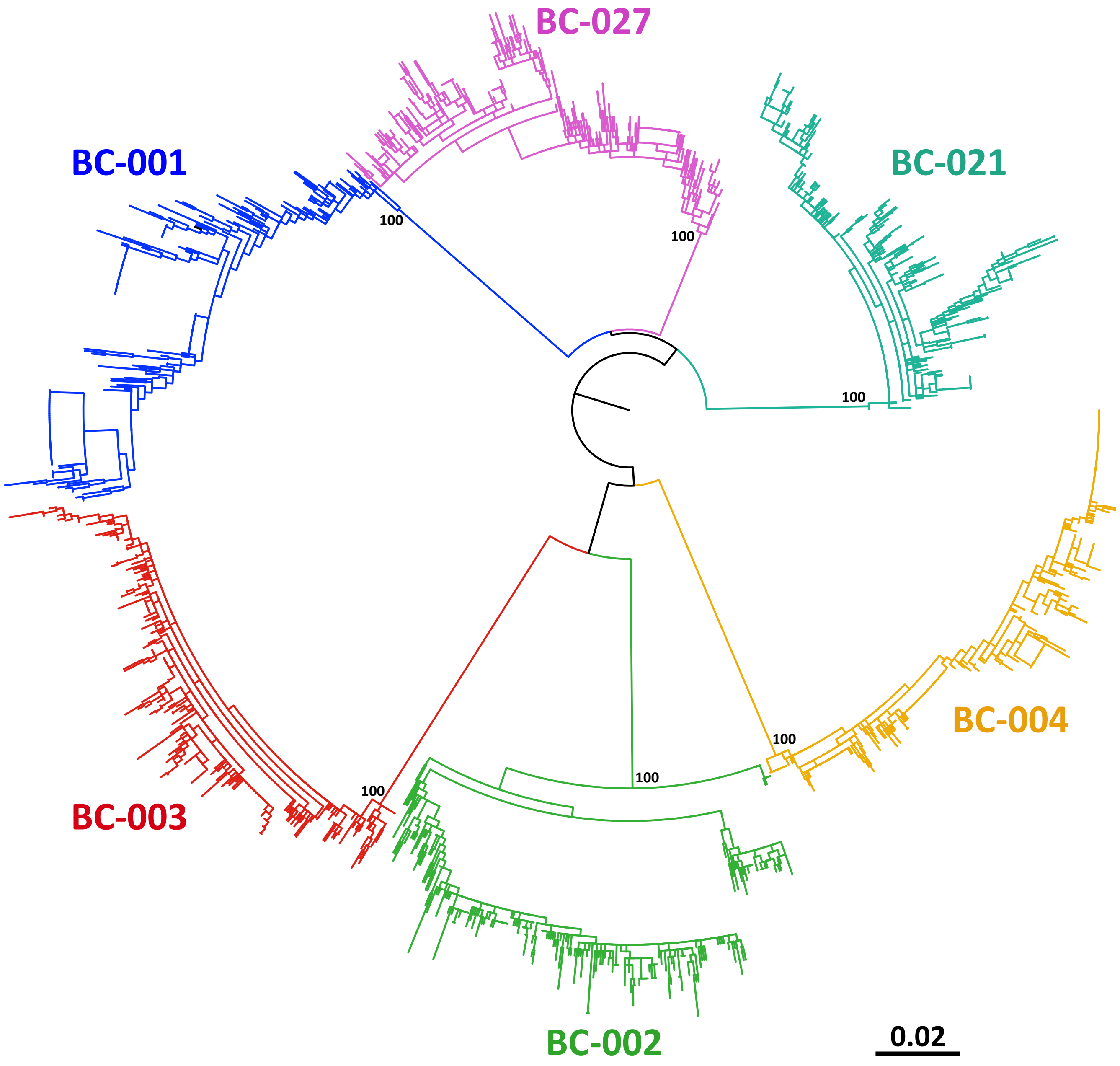

Fig. S1: Between-host phylogeny inferred from *nef* sequence alignments. Maximum-likelihood phylogeny inferred from all pre-ART plasma HIV RNA sequences and all on-ART sequences with an intact *nef* gene. Phylogeny is mid-point rooted. Numbers on internal branches indicate bootstrap values. Scale in estimated substitutions per nucleotide site.

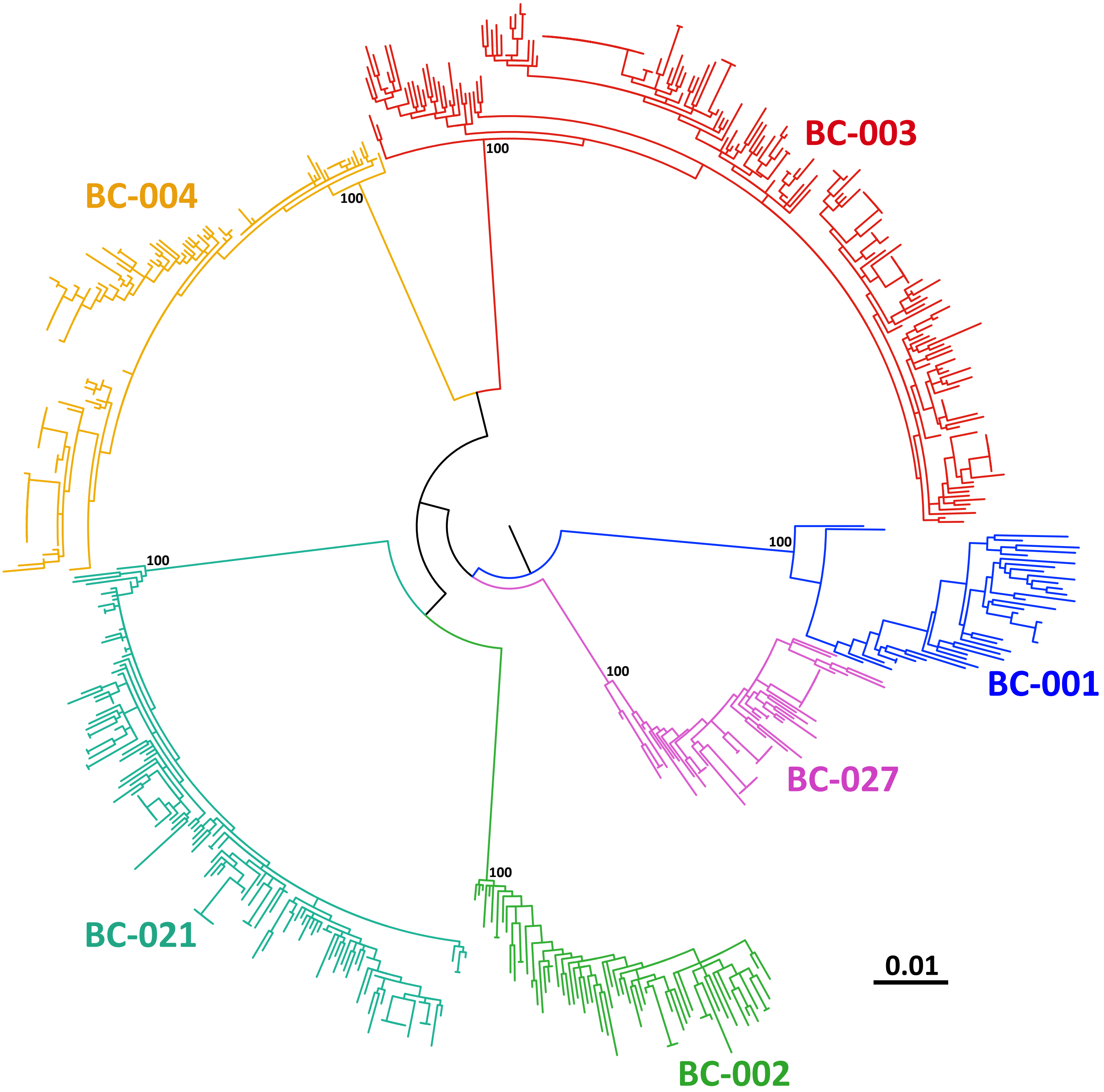

Fig. S2: Between-host phylogeny inferred from *gag* sequence alignments. Same as Figure S1, except this phylogeny was inferred from all on-ART proviral sequences with an intact *gag* gene.

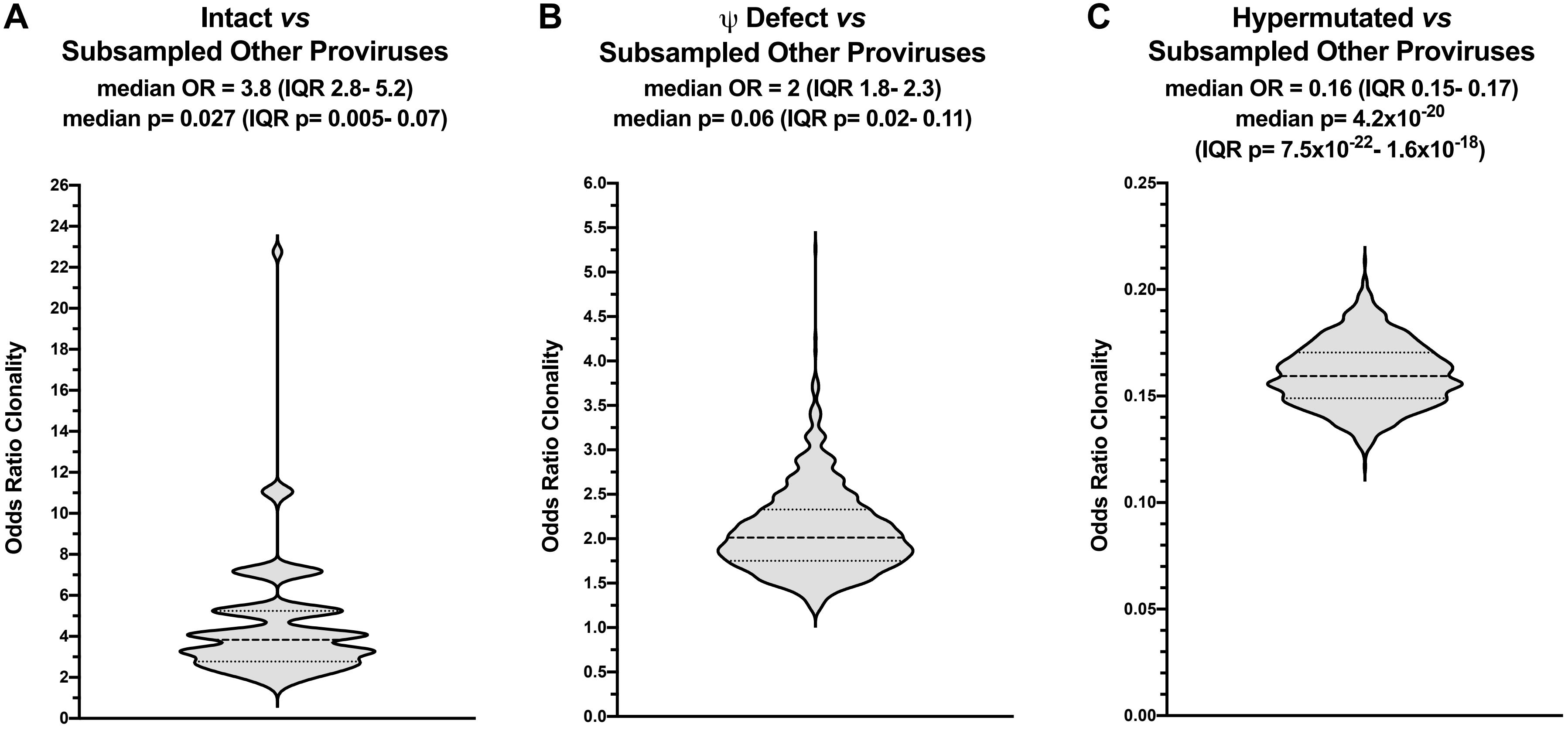

Fig. S3: Odds ratios of clonality by genomic integrity: subsampling analysis. This analysis tests the robustness of significant between-group comparisons in Figures 2B-E to shallower sampling depth. *Panel A.* Odds ratio distributions derived by comparing the clonality of 54 unique intact proviruses with 1,000 equally-sized datasets of defective proviruses, subsampled from the overall data with replacement. *Panel B.* Same as *A*, but comparing the clonality of 94 unique Ψ-defect sequences to 1,000 equally-sized subsamples of proviruses of other types. P*anel C.* Same as *A*, but comparing the clonality of 570 unique hypermutated sequences to 1,000 equally-sized subsamples of proviruses of other types. Odds ratios and p-values were computed using Fisher's exact test and are not corrected for multiple comparisons. On the violin plots, the middle dashed line indicates the median Odds Ratio, while the upper and lower dashed lines denote the 25th and 75th percentiles.

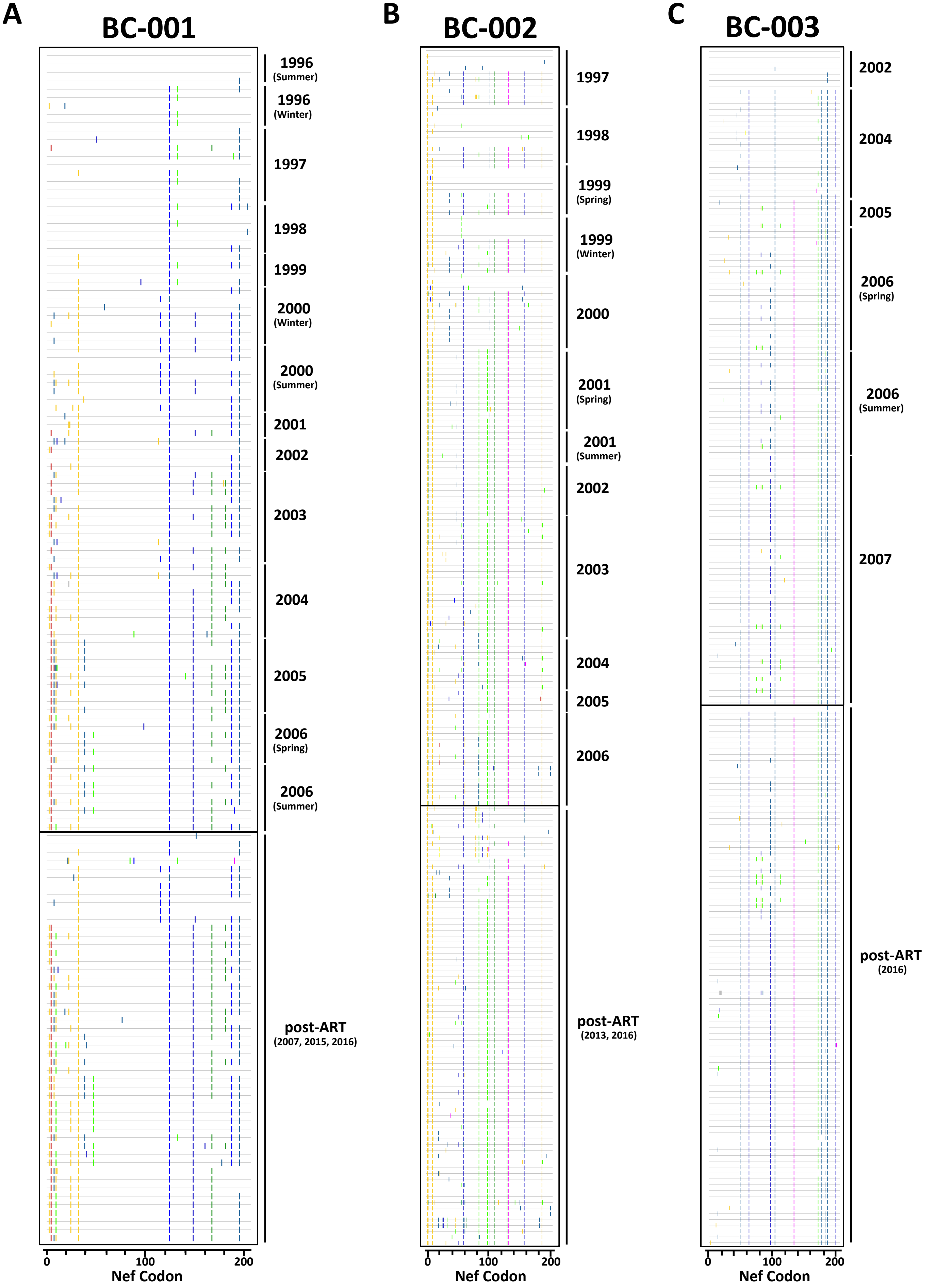

A
BC-001
1996 (Summer)
1996 (Winter)
1997
1998
1999
2000 (Winter)
2000 (Summer)
2001
2002
2003
2004
2005
2006 (Spring)
2006 (Summer)
post-ART (2007, 2015, 2016)
0
100
200
Nef Codon
B
BC-002
1997
1998
1999 (Spring)
1999 (Winter)
2000
2001 (Spring)
2001 (Summer)
2002
2003
2004
2005
2006
post-ART (2013, 2016)
0
100
200
Nef Codon
C
BC-003
2002
2004
2005
2006 (Spring)
2006 (Summer)
2007
post-ART (2016)
0
100
200
Nef Codon

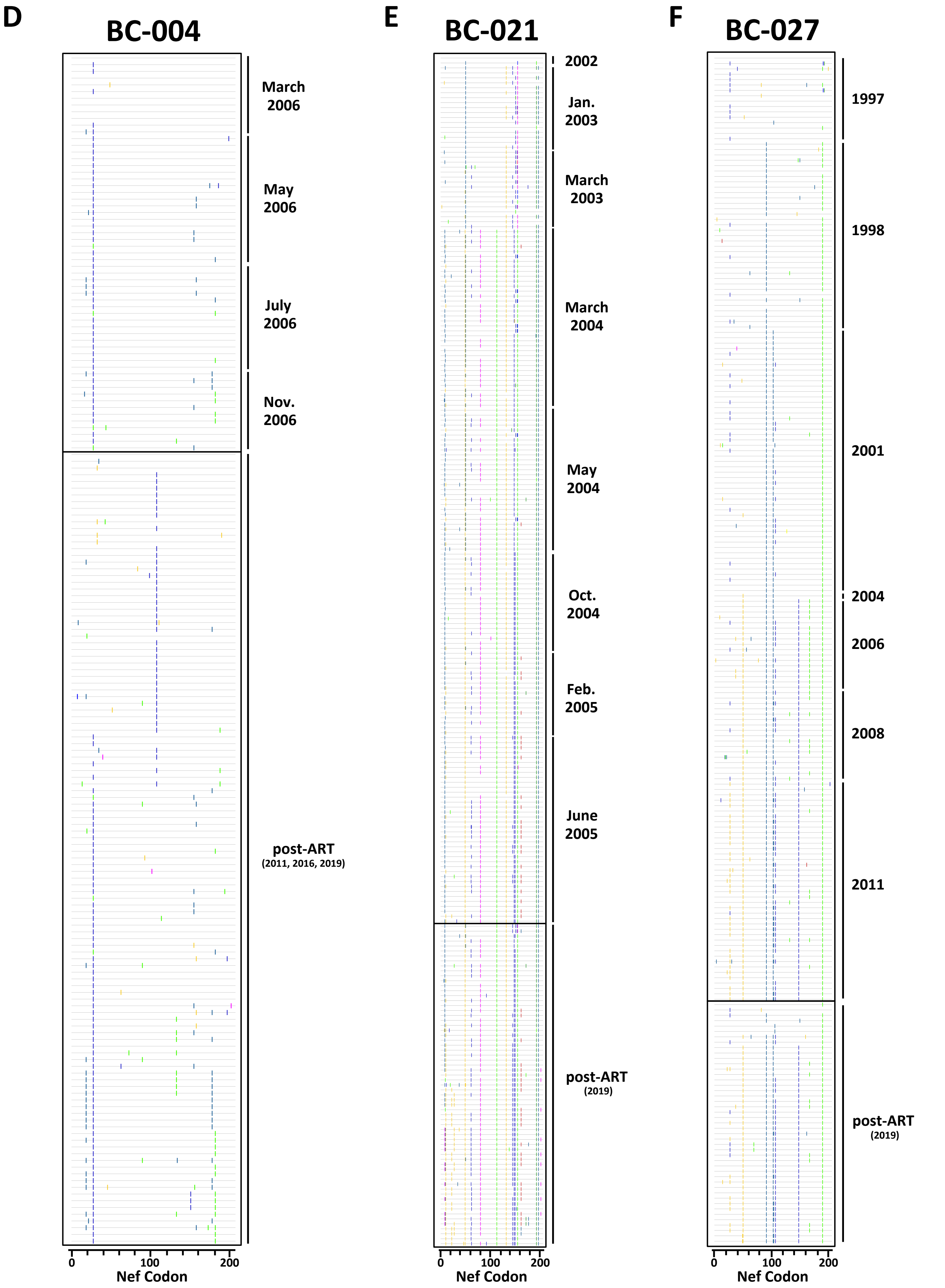
D
BC-004
March 2006
May 2006
July 2006
Nov. 2006
post-ART
(2011, 2016, 2019)
0
100
200
Nef Codon
E
BC-021
2002
Jan. 2003
March 2003
March 2004
May 2004
Oct. 2004
Feb. 2005
June 2005
post-ART
(2019)
0
100
200
Nef Codon
F
BC-027
1997
1998
2001
2004
2006
2008
2011
post-ART
(2019)
0
100
200
Nef Codon

Fig. S4 (previous two pages): Amino acid highlighter plots depicting within-host evolution in pre- and post-ART *nef* sequences for each participant. The top sequence corresponds to the pre-ART plasma sequence closest to the root of the participant's highest likelihood within-host phylogeny (see Figures 3B-8B in main manuscript) and serves as a reference sequence, where colored ticks in sequences beneath this denote non-synonymous substitutions relative to this reference. Pre-ART *nef* sequences are ordered according to their sampling date; post-ART *nef* sequences are ordered according to their inferred integration date.

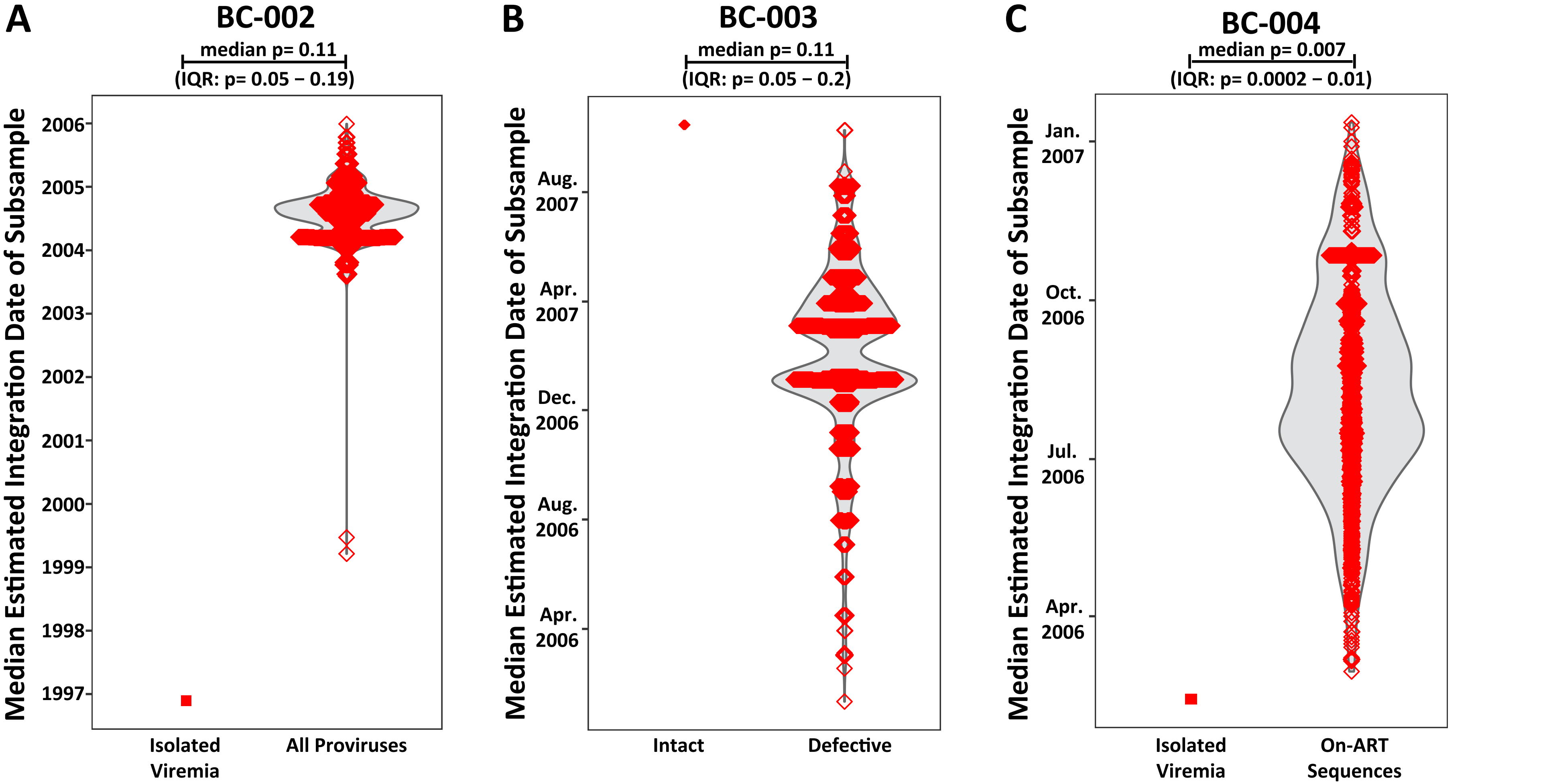

Fig. S5: Comparison of integration date distributions of select groups of sequences: subsampling analysis. This analysis tests the robustness of significant between-group comparisons reported in Figures 4D, 5D and 6D, to shallower sampling depth. *Panel A.* Median integration dates of 9 unique isolated viremia sequences from BC-002 (solid red square) versus those derived from 1,000 equally-sized provirus datasets, subsampled with replacement from the participant's full provirus sequence dataset (open diamonds). *Panel B.* Median integration dates of 15 unique intact proviruses from BC-003 versus those from 1,000 equally-sized subsampled defective provirus datasets. *Panel C.* Median integration dates of 8 unique isolated viremia sequences from BC-004 versus those from 1,000 equally-sized subsampled on-ART sequences. The median (and IQR) p-values from all 1,000 comparisons are shown above each plot.

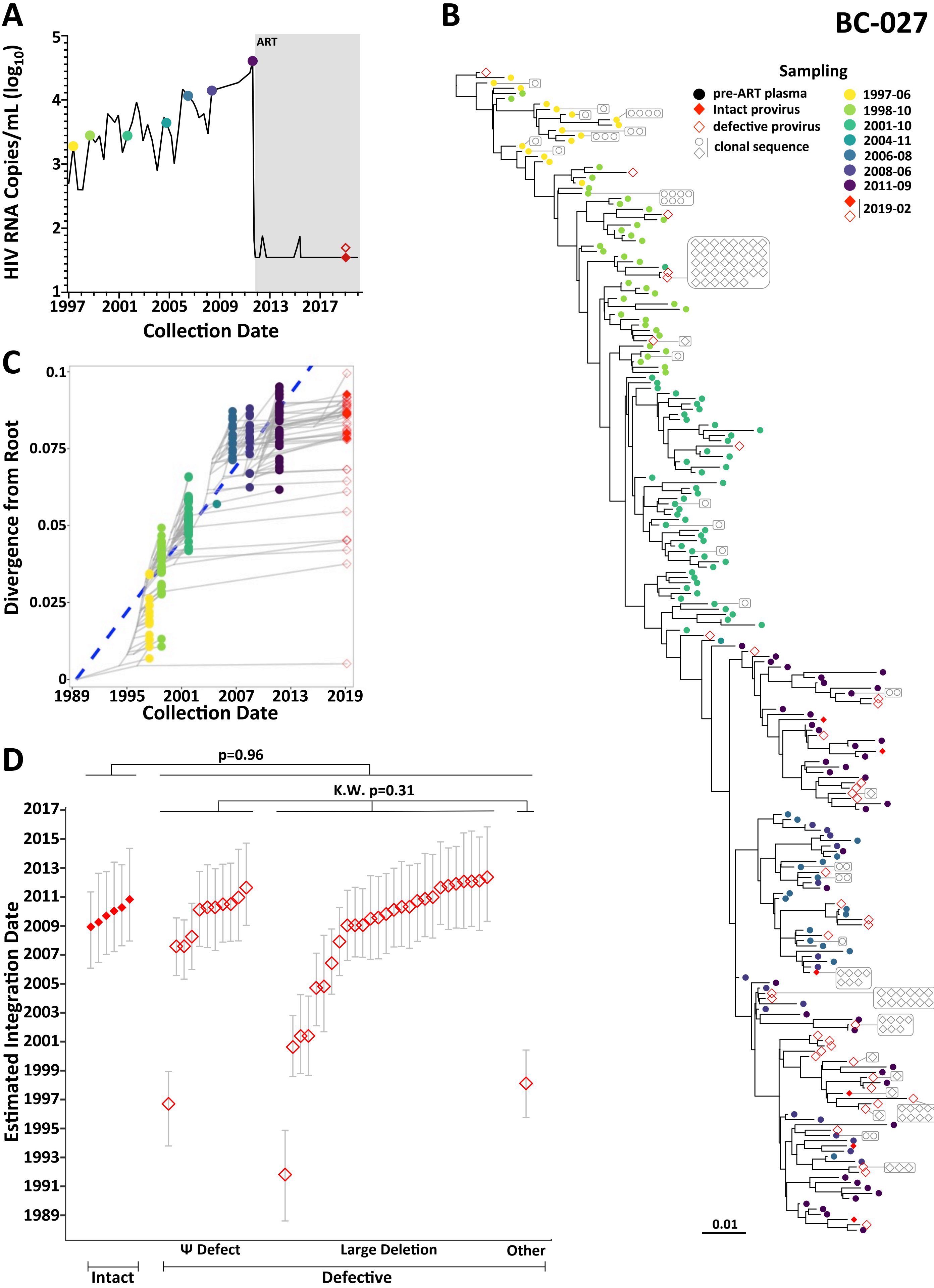

A
B
BC-027
ART
HIV RNA Copies/mL (log_{10})
Collection Date
Sampling
pre-ART plasma
Intact provirus
defective provirus
clonal sequence
1997-06
1998-10
2001-10
2004-11
2006-08
2008-06
2011-09
2019-02
0.01
C
Divergence from Root
Collection Date
D
p=0.96
K.W. p=0.31
Estimated Integration Date
Ψ Defect
Large Deletion
Other
Intact
Defective

Fig. S6 (previous page): Integration date inference of on-ART sequences for participant BC-027, after out-group rooting their phylogenies. The data, analyses and legend are the same as for Figure 8, except that all of the phylogenies in the present analysis were out-group rooted, and this root was used to infer integration dates of on-ART proviruses.

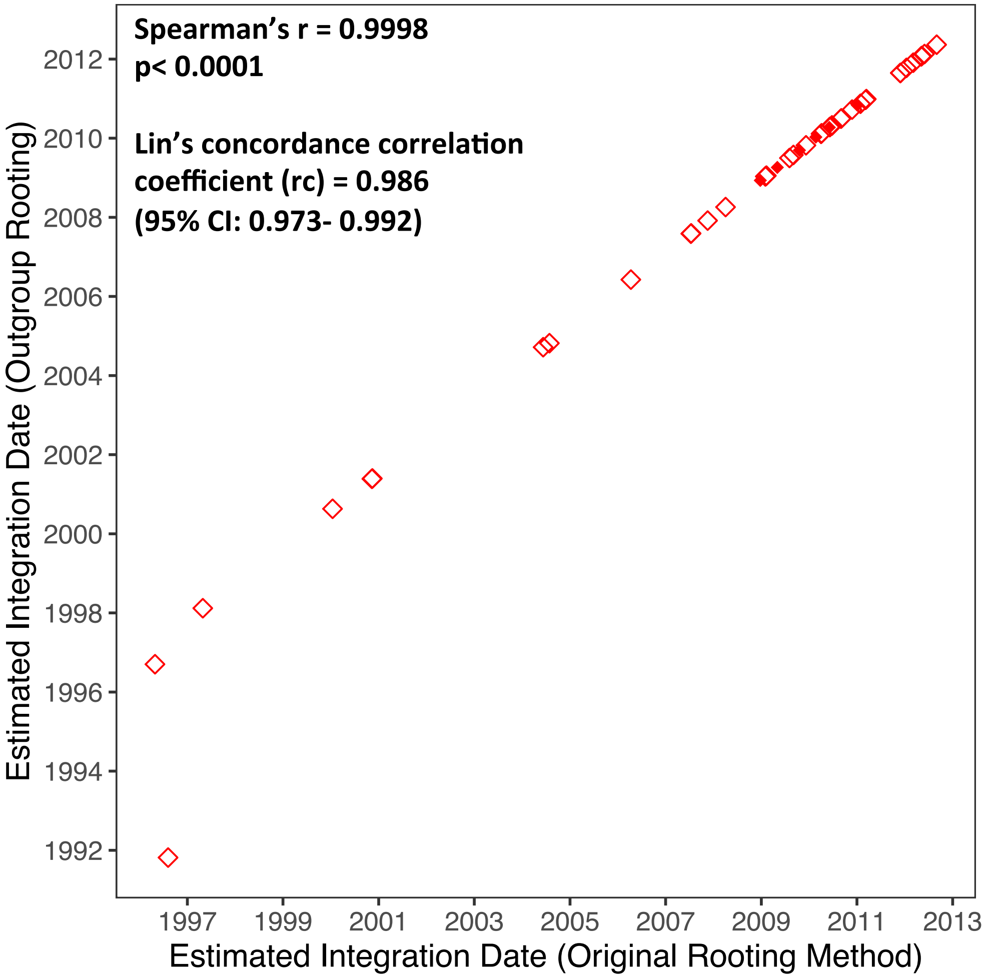

Fig. S7: Relationship between original versus out-group-root-derived proviral integration dates for participant BC-027.

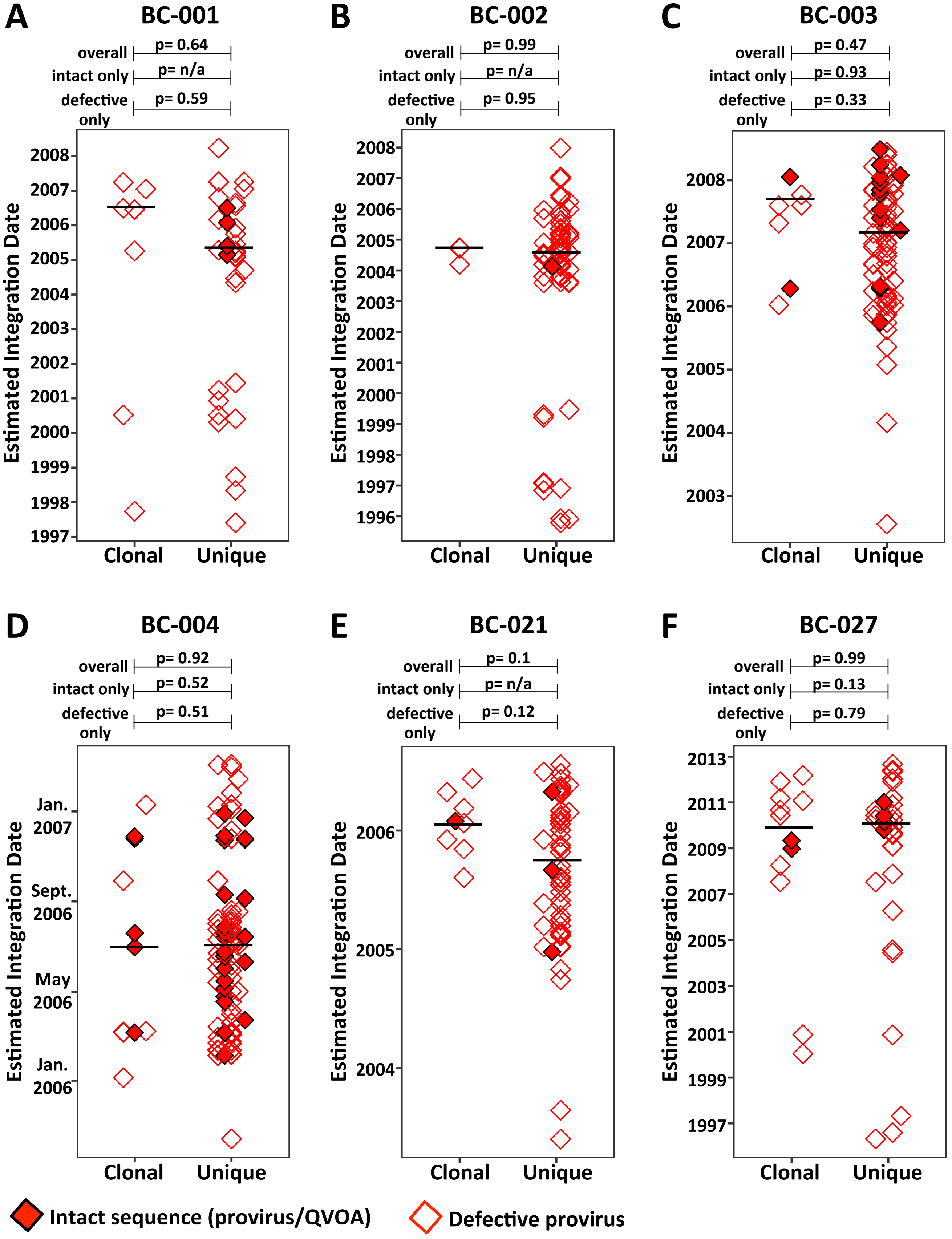
A
BC-001
overall p= 0.64
intact only p= n/a
defective only p= 0.59
Estimated Integration Date
2008
2007
2006
2005
2004
2003
2002
2001
2000
1999
1998
1997
Clonal
Unique
B
BC-002
overall p= 0.99
intact only p= n/a
defective only p= 0.95
Estimated Integration Date
2008
2007
2006
2005
2004
2003
2002
2001
2000
1999
1998
1997
1996
Clonal
Unique
C
BC-003
overall p= 0.47
intact only p= 0.93
defective only p= 0.33
Estimated Integration Date
2008
2007
2006
2005
2004
2003
Clonal
Unique
D
BC-004
overall p= 0.92
intact only p= 0.52
defective only p= 0.51
Estimated Integration Date
Jan. 2007
Sept. 2006
May 2006
Jan. 2006
Clonal
Unique
E
BC-021
overall p= 0.1
intact only p= n/a
defective only p= 0.12
Estimated Integration Date
2006
2005
2004
Clonal
Unique
F
BC-027
overall p= 0.99
intact only p= 0.13
defective only p= 0.79
Estimated Integration Date
2013
2011
2009
2007
2005
2003
2001
1999
1997
Clonal
Unique
Intact sequence (provirus/QVOA)
Defective provirus

Fig. S8 (previous page): Lack of relationship between sequence clonality and age. Estimated integration dates of distinct proviruses that were observed two or more times (clonal) versus only once (unique), by participant. Open diamonds denote defective proviruses; red diamonds with black outline include intact proviruses as well as HIV RNA sequences recovered from *ex vivo* reactivation (QVOA), if any. Black lines indicate median estimated integration date for each group. P-values were determined using the Mann-Whitney test, and are not corrected for multiple testing.
