# Supplementary material for: HIV reservoirs are dominated by genetically younger and clonally enriched proviruses
Source: mBio. 2023 Nov 16;14(6):e02417-23. doi: 10.1128/mbio.02417-23 (PMC10746175; doi:10.1128/mbio.02417-23)
Supplement: Table S1 — Participant reservoir composition and size. [file mbio.02417-23-s0002.docx]

|  | BC-001 | BC-002 | BC-003 | BC-004 | BC-021 | BC-027 | Total |
| --- | --- | --- | --- | --- | --- | --- | --- |
| Total proviral sequences (n) | 317 | 265 | 733 | 440 | 386 | 195 | 2,336 |
| Intact (n) | 5 | 1 | 15 | 47 | 9 | 14 | 91 |
| Total defective (n) | 312 | 264 | 718 | 393 | 377 | 181 | 2,245 |
| Hypermutated (n) | 95 | 34 | 386 | 107 | 7 | 7 | 636 |
| Ψ defective (n) | 68 | 15 | 34 | 46 | 15 | 44 | 222 |
| Large deletion (n) | 138 | 201 | 285 | 227 | 349 | 129 | 1,329 |
| Inversion (n) | 4 | 9 | 3 | 7 | 2 | 1 | 26 |
| Premature stop (n) | 2 | 4 | 6 | 4 | 3 | - | 19 |
| Scramble (n) | 1 | 1 | 3 | - | 1 | - | 6 |
| HIV-Human chimera (n) | 4 | - | 1 | 2 | - | - | 7 |
| Total HIV Copies (per Million CD4+ T-cells)^a^ | 1,757 | 4,052 | 1,930 | 1,851 | 765 | 518 |  |
| Intact HIV Copies (per Million CD4+ T-cells)^a^ | 85 | 199 | 238 | 160 | 70 | 32 |  |
| Defective HIV Copies (per Million CD4+ T-cells)^a^ | 1,672 | 3,853 | 1,692 | 1,691 | 695 | 486 |  |

**Table S1: Participant** **reservoir composition and size**

^a^ measured using the Intact Proviral DNA Assay (IPDA)
