# Supplementary material for: HIV reservoirs are dominated by genetically younger and clonally enriched proviruses
Source: mBio. 2023 Nov 16;14(6):e02417-23. doi: 10.1128/mbio.02417-23 (PMC10746175; doi:10.1128/mbio.02417-23)
Supplement: Table S2 — Bayesian within-host phylogenetic inference details. [file mbio.02417-23-s0003.docx]

**Table S2**: **Bayesian within-host phylogenetic inference details**

| **Participant^a^** | **MCMC generations**  **(Million)** | **Post**  **burn-in phylogenies** | **QC-passed**  **phylogenies**  N (%) | Δ**AIC^bc^**  **(median, IQR)** | **Phylogenetically**  **estimated root date (95% HPD^d^)** | **Within-host HIV**  **evolutionary rate^e^ (mean, 95%HPD)** |
| --- | --- | --- | --- | --- | --- | --- |
| BC-001 | 10 | 1,500 | 1,500 (100%) | 150 (131- 164) | Feb-1995 (Dec-1993 –Feb-1996) | 3x10^-5^ (1.7x10^-5^ – 4.2x10^-5^) |
| BC-002 | 10 | 1,500 | 1,500 (100%) | 106 (89- 121) | May-1992 (Aug-1989 –Oct- 1994) | 1.5x10^-5^ (8.2x10^-6^ – 2.2x10^-5^) |
| BC-003 | 30 | 4,500 | 4,500 (100%) | 137 (120- 156) | Dec-2001 (Feb-2001 -Aug-2002) | 6.8x10^-5^ (4x10^-5^ – 1x10^-4^) |
| BC-004 | 40 | 6,000 | 1,622 (27%) | 14 (11- 17) | Apr-2005 (Oct-2004 -Sept-2005) | 1x10^-4^ (4.7x10^-5^ – 1.6x10^-4^) |
| BC-021 | 20 | 3,000 | 3,000 (100%) | 263 (239- 282) | Apr-2002 (Dec-2001 – Aug-2002) | 8.4x10^-5^ (5.1x10^-5^ – 1.2x10^-4^) |
| BC-027 | 10 | 1,500 | 1,500 (100%) | 330 (292- 366) | Sept-1992 (Jan-1990 – Mar-1995) | 1.5x10^-5^ (9.1x10^-6^ – 2.1x10^-5^) |

^a^ The best-fitting nucleotide substitution model for BC-002 was HKY+I+G; for all other participants it was GTR+I+G (see methods).

^b^ ΔAIC, delta Akaike information criterion. Median and IQR represent that of all phylogenies that passed quality control (QC; see methods).

^c^ The ΔAICs reported here translate to a median Pearson R of 0.85 (IQR 0.79- 0.88, range 0.43- 0.97) (p-value range 0- 0.007).

^d^ 95% HPD, 95% highest posterior density.

^e^ Expressed in estimated substitutions per nucleotide site per day.
